# Modeling-Dependent Protein Characterization of the Rice Aldehyde Dehydrogenase (ALDH) Superfamily Reveals Distinct Functional and Structural Features

**DOI:** 10.1371/journal.pone.0011516

**Published:** 2010-07-12

**Authors:** Simeon O. Kotchoni, Jose C. Jimenez-Lopez, Dongying Gao, Vincent Edwards, Emma W. Gachomo, Venu M. Margam, Manfredo J. Seufferheld

**Affiliations:** 1 Department of Agronomy, Purdue University, West Lafayette, Indiana, United States of America; 2 Department of Biochemistry, Cell and Molecular Biology of Plants, Estacion Experimental del Zaidin, Consejo Superior de Investigaciones Cientificas, Granada, Spain; 3 Department of Biology and Microbiology, South Dakota State University, Brookings, South Dakota, United States of America; 4 Department of Entomology, Purdue University, West Lafayette, Indiana, United States of America; 5 Department of Crop Science, University of Illinois Urbana-Champaign, Urbana, Illinois, United States of America; University of Pennsylvania, United States of America

## Abstract

The completion of the rice genome sequence has made it possible to identify and characterize new genes and to perform comparative genomics studies across taxa. The aldehyde dehydrogenase (ALDH) gene superfamily encoding for NAD(P)^+^-dependent enzymes is found in all major plant and animal taxa. However, the characterization of plant ALDHs has lagged behind their animal- and prokaryotic-ALDH homologs. In plants, ALDHs are involved in abiotic stress tolerance, male sterility restoration, embryo development and seed viability and maturation. However, there is still no structural property-dependent functional characterization of ALDH protein superfamily in plants. In this paper, we identify members of the rice ALDH gene superfamily and use the evolutionary nesting events of retrotransposons and protein-modeling–based structural reconstitution to report the genetic and molecular and structural features of each member of the rice ALDH superfamily in abiotic/biotic stress responses and developmental processes. Our results indicate that rice-ALDHs are the most expanded plant ALDHs ever characterized. This work represents the first report of specific structural features mediating functionality of the whole families of ALDHs in an organism ever characterized.

## Introduction

Aldehydes are intermediates in several fundamental metabolic pathways, including the syntheses of carbohydrates, vitamins, steroids, amino acids and lipids [1], [2]. They are also produced in response to environmental stresses, including salinity, dehydration, desiccation, cold, and heat shock [3]–[5]. Aldehyde molecules are chemically reactive; at excessive physiological concentrations they are toxic and negatively impact cell growth, yield and seed survival [5]–[7]. Therefore, aldehyde levels in cells must be tightly regulated.

Aldehyde dehydrogenases are an evolutionarily conserved group of enzymes that catalyze the irreversible oxidation of a wide range of endogenous reactive aldehyde molecules to their corresponding carboxylic acids [4], [5], [8]. These include the substrate-specific; the non-substrate specific ALDHs; the betaine dehydrogenases; the non-phosphorylating glyceraldehyde 3-phosphate dehydrogenases; the phenylacetaldehyde dehydrogenases; the lactaldehyde dehydrogenases and the ALDH-like proteins [8]. They are functionally well characterized in bacteria, humans, fungi, and metazoa [8]. ALDH1A1 has been described as an androgen-binding protein in human genital fibroblasts, a thyroid hormone-binding protein in Xenopus liver and a sterol-binding protein in bovine lens epithelial cells, while ALDH2 has been characterized as an acetaminophen- and 1,3-dinitrobenzene-binding protein [8]. However, functional and structural characterizations of plant ALDHs and gene duplication events underlying their diversification have lagged behind that of their mammalian and bacterial counterparts.

Several lines of evidence support the idea that plant ALDHs play crucial roles in development, growth and stress responses [5], [7], [9]. In maize, ALDH2B2 (also known as rf2) has been characterized as a nuclear restorer [10], while the antiquitin ALDH7A1 is a regulator of turgor pressure and functions in general plant stress responses [11]. Loss of ALDH7 function in rice endosperm leads to seed browning during seed desiccation and storage, suggesting that OsALDH7 is critical for seed maturation [7]. Recently, we and several other groups demonstrated that selected members of the ALDH gene superfamily might be critical in plant responses to a wide range of environmental stresses [9], [12]. Ectopic expression of *ALDH3I1* and *ALDH7B4* genes in plants was sufficient to enhance tolerance to drought, salinity and oxidative stress [5], [13]. The OsALDH2 gene, which is induced under submerged stress conditions [14], was up-regulated by stresses and ABA in young leaves. The resurrection plant *Craterostigma plantagineum* (Scrophulariaceae) is a desiccation-tolerant plant that can withstand almost complete water loss and recover within hours after rehydration [3]. The expression of many genes has recently been implicated in the complex desiccation-tolerant trait of *C. plantagineum*, and ALDH3 (CpALDH) was strongly expressed upon and throughout desiccation of the plant [15]. These studies indicate the importance of active ALDHs as genetic tools to engineer crops with enhanced tolerance to environmental stress conditions.

In many species with completely sequenced genomes, a significant amount of genetic information of novel ALDHs has been obtained. Although the fully sequenced plant genomes currently include *Arabidopsis thaliana* (TAIR, http://www.arabidopsis.org/), *Oryza sativa* [16] and *Zea mays* [17], *A. thaliana* is the only plant for which the ALDH gene superfamily has been fully characterized [18] according to the ALDH Gene Nomenclature Committee (AGNC). Specific criteria for cataloging/characterizing ALDH proteins have been established by the AGNC [2]. Based upon these criteria, protein sequences with more than 40% identity to a previously identified ALDH sequence represent a family, and sequences with more than 60% identity within the ALDH family represent a protein subfamily. Unlike the comprehensive study of human ALDHs [1], [19], a unified plant ALDH nomenclature has not been established except for Arabidopsis [18]. Recently, Gao and Han [20] described the evolution of the rice ALDH gene superfamily. However, their work did not attempt to revise the gene nomenclature according to the standardized AGNC-accepted criteria. In addition, the rice ALDH gene superfamily reported by Gao and Han [20] is incomplete. Here we report a complete list of the rice ALDH genes, and we present a revised and unified nomenclature for the rice ALDHs based on the AGNC criteria.

Rice (*O. sativa*) is an important food crop and a model crop plant for studying monocots. This economically valuable crop has suffered significant yield losses due to drought and a combination of other environmental stresses; therefore, developing stress-tolerant rice varieties is vital for agricultural sustainability.

Although pieces of evidence suggest that the rice ALDHs could be used for crop improvement, relatively little is known about their 3D structural features and the molecular properties of their NAD-ring binding clefts in plants. In this paper, we take advantage of the completely sequenced rice genome (International Rice Genome Sequencing Project 2005) to provide for the first time a revised annotation for the rice *ALDH* gene superfamily based upon the unified nomenclature criteria developed by AGNC. Also, we examined the chronological events of all rice ALDH transposable elements. In addition, we employ a phylogenetic analysis tool and a computational modeling approach to study the structural/molecular conformational features of each class of the rice ALDH superfamily, and provide a comparative functional analysis with previously well-characterized plant ALDHs.

## Results

### The rice *ALDH* gene superfamily: revised nomenclature and phylogenetic analysis

The completion of the rice genome sequencing project paved the way for gene discovery, functional gene analyses and comparative genomics studies using the rice gene data. We searched the entire rice genome sequence for deduced amino acid sequences similar to those of previously characterized ALDHs, identified corresponding rice ALDHs and assigned them to different ALDH protein families based on the AGNC criteria (Table 1). To retrieve the rice ALDHs, we used the conserved ALDH motifs, the conserved active sites, the defined family criteria (as detailed in the Materials and Methods), and the Arabidopsis *ALDH* gene superfamily [18] as database entry-points for search queries. We then carried out a validation database search using the annotated rice genome database [16] in which only full-length (FL) rice cDNAs with high (∼98%) matches to candidate *ALDH* sequences were considered. We verified all annotated rice ALDH open reading frames (ORFs) by comparing them with cDNA and EST sequences.

**Table 1:** The rice ALDH protein superfamily: revised nomenclature.

ALDH Family	Revised Annotation	Gene Locus	Molecular Function	Subcellular Localization	CDS (bp)	Num. A.A.	M.W. (kDa)
Family 2	OsALDH2B1	Os06g15990	Aldehyde dehydrogenase	Mitochondrion	1650	549	59.3
	OsALDH2B2	Os06g39230	Aldehyde dehydrogenase	Cytosol	1581	526	56.4
	OsALDH2B5	Os02g49720	Aldehyde dehydrogenase	Mitochondrion	1662	553	58.9
	OsALDH2C1	Os01g40870	Aldehyde dehydrogenase	Cytosol	1524	507	54.2
	OsALDH2C4	Os01g40860	aldehyde dehydrogenase (NAD) coniferyl-aldehyde dehydrogenase	Cytosol	1509	502	54.2
Family 3	OsALDH3B1	Os04g45720	Variable substrate ALDH		1500	499	54.3
	OsALDH3E1	Os02g43194	aldehyde dehydrogenase [NAD(P)+]	Chloroplast	1464	487	54.5
	OsALDH3E2	Os02g43280	Variable substrate ALDH	Chloroplast	1476	491	54.6
	OsALDH3H1	Os12g07810	aldehyde dehydrogenase [NAD(P)+]	endoplasmic reticulum, membrane, vacuole	1455	484	52.4
	OsALDH3H2	Os11g08300	Variable substrate ALDH		1449	482	52.5
Family 5	OsALDH5F1	Os02g07760	SSADH, oxidoreductase activity, acting on the aldehyde or oxo group of donors, NAD or NADP as acceptor	Mitochondrion	1584	527	56.1
Family 6	OsALDH6B2	Os07g09060	MM-ALDH	Mitochondrion	1605	534	57.2
Family 7	OsALDH7B6	Os09g26880	Antiquitin		1530	509	54.5
Family 10	OsALDH10A5	Os04g39020	BADH		1518	505	54.6
	OsALDH10A8	Os08g32870	BADH	Chloroplast, plastids	1512	503	54.7
Family 11	OsALDH11A3	Os08g34210	GAPN	Cytoplasm	1500	499	53.4
Family 12	OsALDH12A1	Os05g45960	P5CDH	Mitochondrion	1653	550	60.5
	OsALDH12B1	Os12g40440	P5CDH	Mitochodrion	2427	808	91.0
Family 18	OsALDH18B1	Os05g38150	P5CS		2151	716	77.7
	OsALDH18B2	Os01g62900	P5CS		2208	735	79.5
Family 22	OsALDH22A1	Os07g48920	Aldehyde dehydrogenase (NAD)	Secretory pathway	1794	597	66.0

Homology-based searches resulted in the identification of 21 unique *OsALDH* sequences that encode members of ten ALDH protein families (Table 1), six of which (ALDH2B1, ALDH2B2, ALDH2B5, and ALDH2C1 of family 2; ALDH3E1 of family 3; ALDH6B1 of family 6; ALDH7B6 of family 7; ALDH10A5 of family 10; and ALDH18B1 of family 18) have been previously identified [8]. Five out of the ten ALDH families are represented by multiple *ALDH* gene members (ALDH2: 5 genes; ALDH3: 5 genes; ALDH10: 2 genes; ALDH12: 2 genes; ALDH18: 2 genes) (Table 1). The remaining five families (5; 6; 7; 11; and 22) are represented by a single *ALDH* gene (Table 1). Of all the well-characterized plant ALDHs, the rice *ALDH* gene superfamily is the most extensive, with 21 genes compared to 20 genes in *Physcomitrella patens* [21], 8 genes in *Chlamydomonas reinhardtii* [21], and 14 genes in *Arabidopsis thaliana* [18].

**Figure 1:**
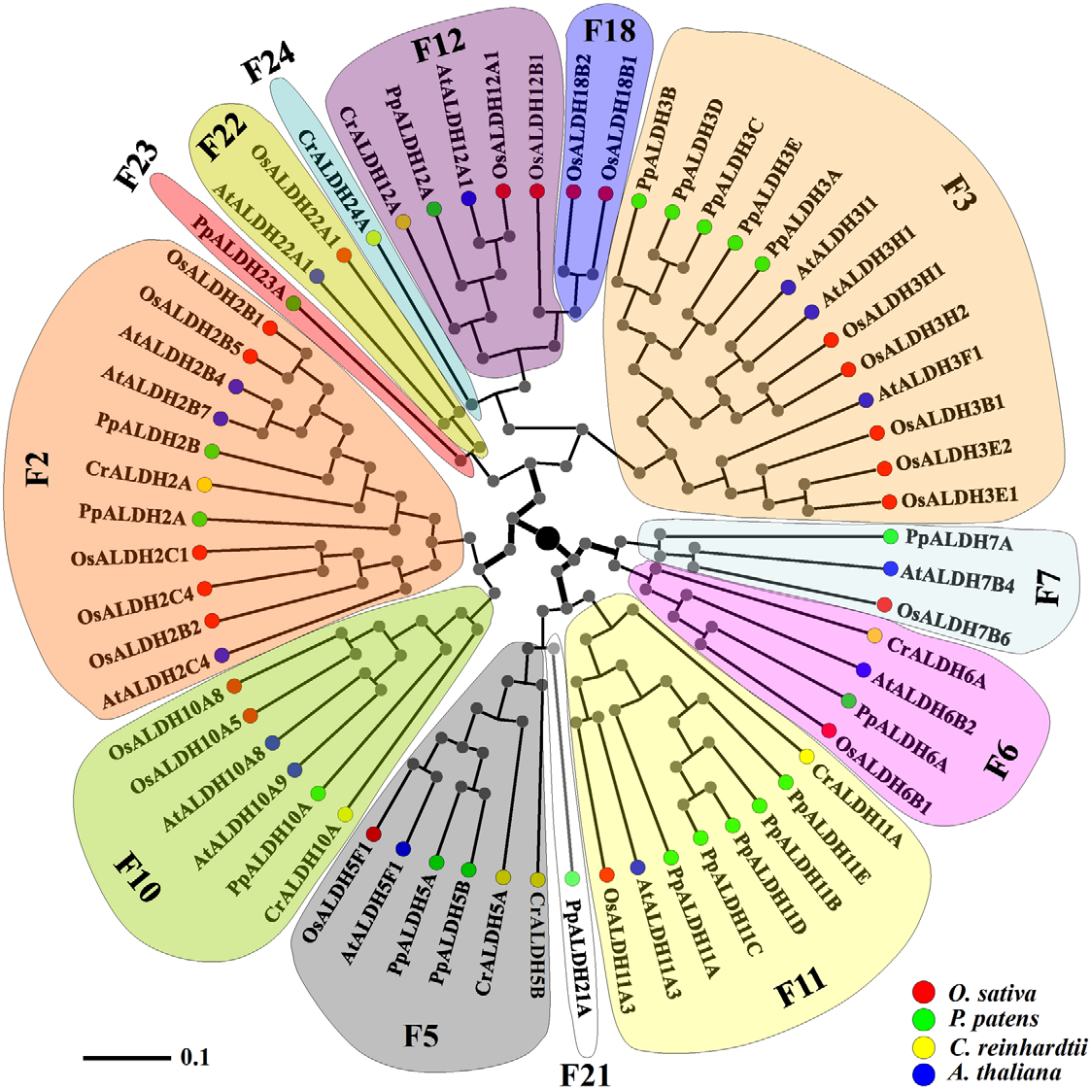
Phylogenetic analysis of well characterized plant ALDHs. Neighbor-Joining (NJ) method was used to performe a phylogenetic analysis of *O. sativa* (red), *A. thaliana* (blue), *P. patens* (green), and *C. reinhardtii* (yellow) deduced ALDH protein sequences. Members of respective ALDH families are depicted in a specific background colour.

In addition to being an important crop, rice is a model system capable of both revealing the genetic evolution of monocots and improving their traits as crops. Although the evolutionary relationships of ALDHs have been the focus of several studies [8], a phylogenetic analysis of rice ALDH sequences with other well-characterized plant ALDHs has never been performed. The resulting phylogenetic tree, which includes well-characterized plant ALDHs and rice ALDHs, is shown in Figure 1. The tree shows that the plant ALDHs are split into four clades, and rice ALDHs share the common core of the plant ALDH families (ALDH2, ALDH3, ALDH5, ALDH6, ALDH7, ALDH10, ALDH11 and ALDH12) with *A. thaliana* and *P. patens* (Figure 1, Table 2). *O. sativa* ALDH sequences are more similar to those of *P. patens* and *A. thaliana* than to *C. reinhardtii* ALDHs, with ADLH23 and ALDH24 found only in *P. patens* (Table 2, [21]) and ALDH22 found in *A. thaliana* and *O. sativa* (Figure 1, Table 2). ALDH22, ADLH23 and ALDH24 are related sequences [21]. For instance, *O. sativa*, *A. thaliana* and *P. patens* have genes that encode ALDH2, ALDH3, ALDH5, ALDH6 and ALDH7, which are present in a wide variety of plants [18]. However, the *C. reinhardtii* genome lacks the ALDH3 and ALDH7 gene families but has the novel gene family ALDH24, which is not present in *O. sativa*, *A. thaliana* or *P. patens* (Figure 1). We found that family 18 (OsALDH18B1 and OsALDH18B2), which encodes P5CS, a crucial enzyme in proline synthesis, is unique to rice (Figure 1). The rice genome has a striking expansion of the ALDH3 and ALDH2 gene families (Figure 1). ALDH2 and ALDH3 contain five genes each, which represent ∼50% of the total number of rice ALDH genes (i.e., 10 genes out of 21) (Table 1). To gain insight into the functional relevance of the more abundant members of the selected classes of ALDHs, we explored the evolutionary events involving retrotransposable elements that nested in the rice *ALDH* genes over several million years ago.

**Table 2:** Comparative identification of the *ALDH* gene families in various organisms.

Organism	ALDH family																							
	1	2	3	4	5	6	7	8	9	10	11	12	13	14	15	16	17	18	19	20	21	22	23	24
***O. sativa***	−	**+**	**+**	−	**+**	**+**	**+**	−	−	**+**	**+**	**+**	−	−	−	−	−	**+**	−	−	−	**+**	−	−
***P. patens***	−	**+**	**+**	−	**+**	**+**	**+**	−	−	**+**	**+**	**+**	−	−	−	−	−	−	−	−	**+**	−	**+**	−
***A. thaliana***	−	**+**	**+**	−	**+**	**+**	**+**	−	−	**+**	**+**	**+**	−	−	−	−	−	−	−	−	−	**+**	−	−
***C. reinhardtii***	−	**+**	−	−	**+**	**+**	−	−	−	**+**	**+**	**+**	−	−	−	−	−	−	−	−	−	−	−	**+**
**Human**	**+**	**+**	**+**	**+**	**+**	**+**	**+**	**+**	**+**	−	−	−	−	−	−	−	−	**+**	−	−	−	−	−	−
**Fungi**	**+**	−	−	**+**	**+**	−	−	−	−	**+**	−	−	−	**+**	**+**	**+**	−	**+**	−	−	−	−	−	−

Presence (+) or absence (−) of *ALDH* gene family is depicted in each indicated organism.

### Evolutionary events of transposable elements nested in rice *ALDH* gene superfamily

**Figure 2:**
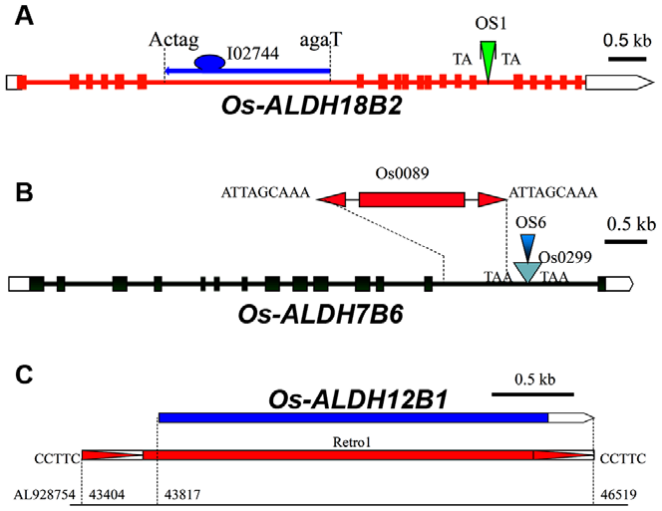
Stress responsive *ALDH* genes are nested by multiple transposable elements. (A) *Os-ALDH18B2* (Os01g62900) gene contains a helitron (I02744) and a MITE (OS1). (B) *Os-ALDH7B6* (Os09g26880) harboured a mutator-like element, Os0089, and a nested MITE block, which includes 2 MITEs. (C) *Os-ALDH12B1* (Os12g40440) gene also serves as a partial sequence of the retrotransposon, Retro1. The capital letters mean TSDs of transposons. The “ctag” and “aga” are 5′ and 3′end sequences of I02744 in Os-ALDH18B2, respectively.

To explore whether transposons are components of the *ALDH* genes in rice, we screened all 21 unique *ALDH* genes using the RepeatMasker program (http://www.repeatmasker.org). Nine out of the twenty-one rice *ALDH* genes contained sixteen transposable elements (TEs) (Table S1). Among the 16 identified TEs, 14 are members of the miniature inverted repeat transposable element (MITE) superfamily (Table S1). Our findings are consistent with previous studies showing that MITEs are preferably inserted into or near genic regions [22], [23]. However, these MITEs were all inserted into introns of the rice *ALDH* genes (Table S1). No MITEs were detected in exons. While the TE evolutionary insertion events are summarized in Table S1, we highlighted the most striking insertion features of the TEs in *ALDH* genes in Figure 2. Two nested MITE blocks were found where a MITE had inserted into another MITE. Some *ALDH* genes harbored more than two TEs; for example, *OsALDH18B2*, which encodes a P5CS enzyme, contained insertions of a helitron (I02744) and a MITE (OS1) (Figure 2A), and the *OsALDH7B6* gene contained three TEs, including a mutator-like element and a nested MITE block (Figure 2B). Intriguingly, the *ALDH12B1* gene was found to act as a partial sequence of a retrotransposon, Retro1 (Figure 2C). The *OsALDH7B6*, *OsALDH12B1* and *OsALDH18B2* genes are known to play essential roles in metabolic processes during plant development and in responses to environmental stresses [5].

To gain insight into the evolution of the *ALDH* gene superfamily, the insertion date of Retro1 was estimated. The results indicate that Retro1 inserted into the genome about 0.43 MYA (million years ago). Since this insertion occurred about 0.43 MYA, the capture of the *OsALDH12B1* sequence and the emergence of the chimeric retrotransposon Retro1 must have occurred more than 0.43 MYA. Our data suggest that the multiple nested TEs in *ALDH* genes have some functional relevance in plant responses to environmental/abiotic stresses, and this feature can be used as a genetic tool to identify and characterize genes that are crucial for stress responses in monocots. For instance, the class 12 ALDHs, which are involved in proline metabolism and Δ1-pyrroline-5-carboxylate (P5C) metabolism, in particular, mediate stress responses and ROS accumulation in plants [24]. Arabidopsis *ALDH12A1*, a P5CDH gene, is highly induced by application of exogenous proline and high salinity [25]. The drought-induced expression of *OsALDH12A1* and *OsALDH12B1* demonstrated that they are potentially involved in rice stress adaptation through proline metabolism [20]. Expression of *ALDH12A1* is regulated by a series of siRNA processing steps during salt stress [24]. Recently, the role of Arabidopsis P5CS1 in stress-induced proline synthesis and the function of P5CS2 in embryo development were characterized in detail [26]. Likewise, members of the class 18 *ALDHs*, which encode P5CS enzymes, are crucial for stress adaptation and salt stress tolerance in rice [27]. These stress-related *ALDH* genes were found to contain multiple TEs (Figure 2, Table S1). We postulated that the striking multiple nested TE events might reflect dynamic evolutionary adaptations to environmental conditions for survival. If so, we expected that all stress-related *ALDH* genes should contain at least two or more TEs. Our previous results demonstrated that classes 3 and 7 of the Arabidopsis ALDHs, including AtALDH3I1 and AtALDH7B4, are crucial for abiotic stress adaptation [5]. We therefore expected that the rice orthologs of the class 3 and class 7 ALDHs would contain multiple TEs. Indeed, our genetic screen found that multiple TEs were nested in the *OsALDH3I1* and *OsALDH7B6* genes as predicted (Figure 2A, Table S1).

### The rice ALDH protein superfamily: structural modeling and functional characterization

**Figure 3:**
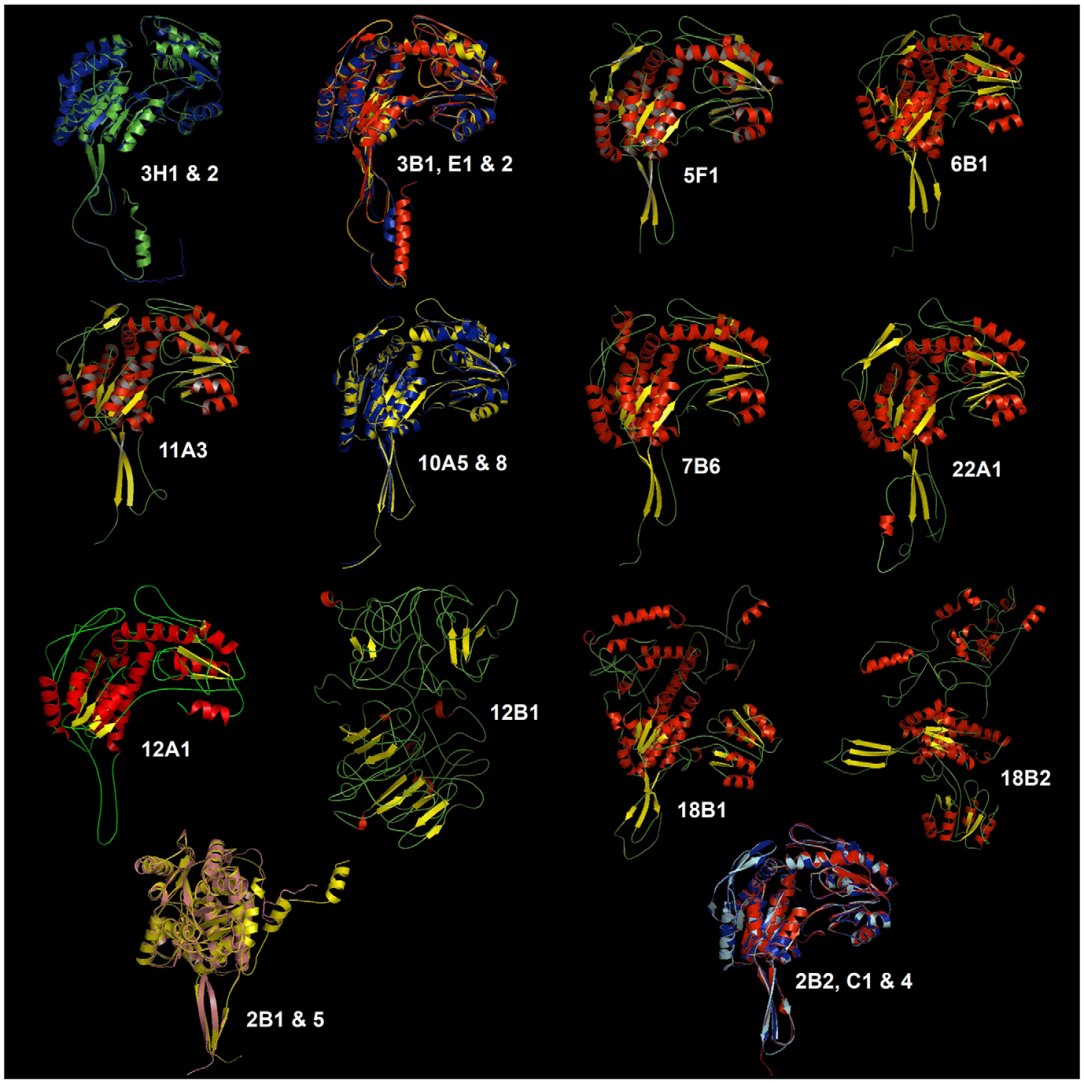
Three-dimensional structure analysis of rice ALDH protein superfamily. All structures are depicted as a cartoon diagram. Within the represented family, the secondary elements are coloured in red (α-helix), yellow (β-sheet) and green (coils). Superimpositions are coloured as follow: Green (ALDH3H1), Blue (ALDH3H2); red (ALDH3B1), yellow (ALDH3E1), blue (ALDH3E2); yellow (ALDH10A5), blue (ALDH10A8); pink (ALDH2B1), yellow (ALDH2B5); red (ALDH2B2), blue (ALDH2C1), turquoise (ALDH2C4).

**Figure 4:**
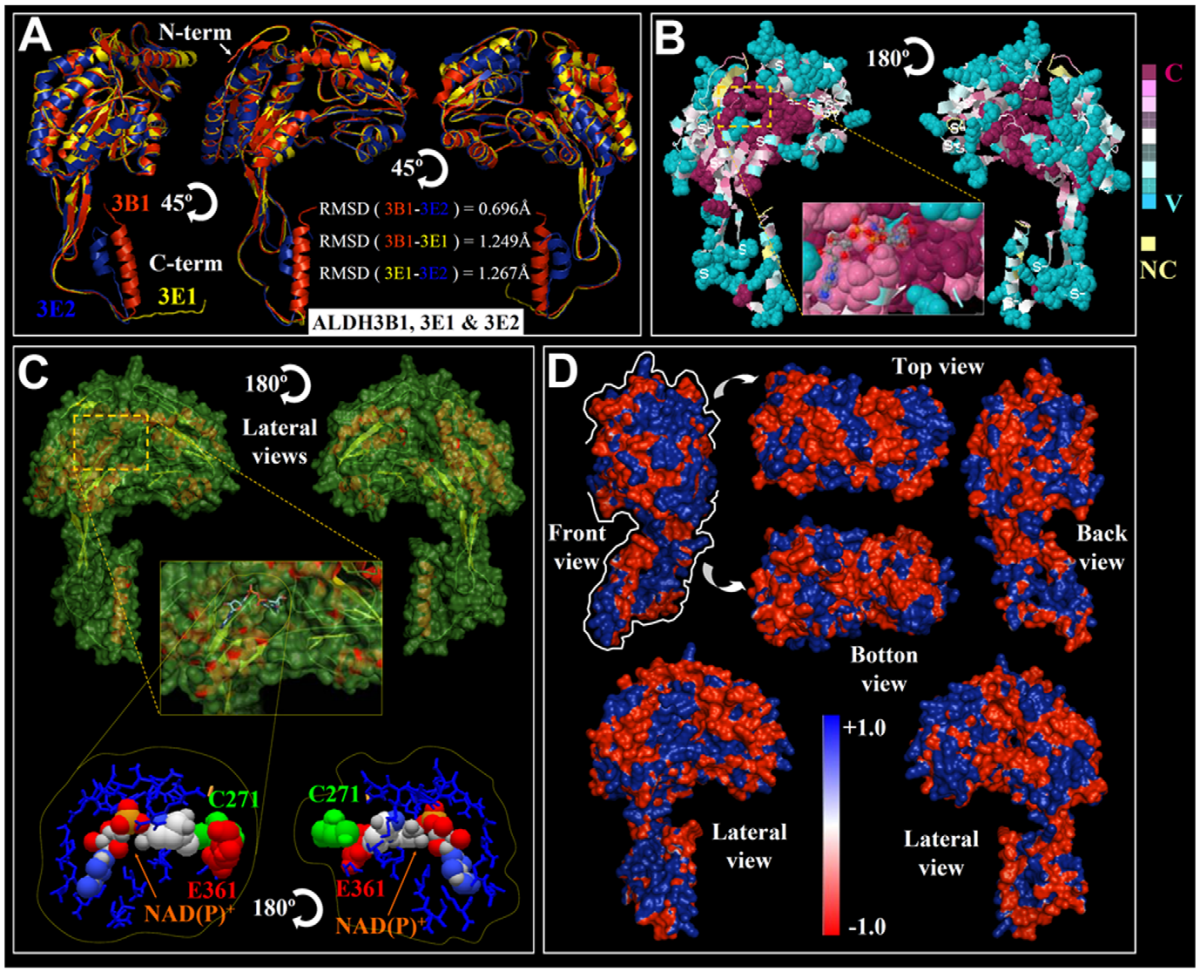
Detailed structural conformation and conservation analysis of selected members of rice ALDH family 3. (A) General structure (cartoon diagram) shows the superimposition of OsALDH3B1 (red), ALDH3E1 (yellow) and ALDH3E2 (blue) with RMSD calculated for each superimposition. Represented structures were rotated at 45°. (B) Best predicted ALDH3B1 model (2D-structure) was subject to consurf-conservational analysis searching for close homologous sequences with known structures using PSI-BLAST. The protein was finally visualized using FirstGlance in Jmol with the conservation scores being colour-coded. The conserved and variable residues are presented as space-filled models and coloured according to the conservation scores. A detailed view of the cavity holding up the NAD(P)^+^ cofactor (stick model and van der Walls spheres) is shown in high magnification. (C) The surface conformation of ALDH3B1 (rotated 180°) showing the secondary structure elements inside is depicted. The morphology of the cavity accommodating NAD(P)^+^ cofactor is represented in high magnification. Detail view organization of the predicted amino acids of the pocket is represented in blue colour. The space-filled representation of van der Waals surface of the cofactor, and the catalytic amino acid residues (Cys 271 in green colour and Glu 361 in red) are opposite positioned. (D) Electrostatic surface potential showing all possible views of ALDH3B1 structure. The surface colours are clamped at red (−1) or blue (+1). Top and bottom views are highlighted with a white line coming from front view.

The ALDH gene superfamily has been characterized in several organisms [8], and the crystallographic structural coordinates of selected ALDHs have been deposited in the Protein Database (PDB) [28]. To our knowledge, structural modeling and conformational feature comparisons of all the members of the ALDH protein superfamily have not been performed in any organism. Using computational modeling, we determined the structural features and uniqueness of the 3D structure of the active sites and the NAD(P)^+^-ring binding clefts of the members of the entire rice ALDH superfamily. Each sequence was modeled based on the ten best structural templates (Figure 3, Figure 4, Figures S1, S2, S3, S4, S5, S6, S7, S8, S9, S10, S11, S12, and S13) using the structural parameters summarized in Table S2. C-scores were used to estimate the quality of the predicted models based on coverage parameters in the structural simulations and alignment with the template. C-score is a confidence scoring function to assessing the quality of a prediction and estimate the accuracy of the I-TASSER predictions, which is defined based on the quality of the threading alignments and the convergence of I-TASSER's structural assembly refinement simulations. Typically, a good predicted model was obtained from a protein sequence when the estimated level of confidence (C-score) was between −5 and 2. The level of confidence of our predicted models for all the rice ALDHs were in the range of −2.26 to 1.75 (Table S2), indicating that the structures were constructed with high accuracy. Because the native structures have not been crystallized, the structural similarity and accuracy of the models were further checked using the TM-score and root mean square deviation (RMSD) parameters. The correct topology of the models was obtained for all structures with TM-scores >0.5, while TM-score values <0.17 indicated that the predicted structure had low accuracy; which was independent of the protein length [29]. Using these parameters, only ALDH18B1, ALDH18B2 and ALDH12B1 had TM-scores equal to or below 0.5 (0.50, 0.46 and 0.45, respectively) and were within the limit of accuracy but with C-scores higher than −5 (Table S2). The low quality of the modeling might be due to a possible divergence of these ALDH families, being members of two separate branches of the same cluster integrating ALDH family 18 and family 12 (Figure 1). General structural comparisons (Figure 3) and phylogenetic analyses (Figure 1) provided clearer and unexpected insight into the structural divergence of the rice ALDHs. Considering the estimated RMSDs (based on the Cα) of all residues in a pairwise comparison of the predicted models in each cluster, we only show representative models for each family or phylogenetic cluster to reduce the number of structural figures (Figure 3, Figure 4, and Figures S1, S2, S3, S4, S5, S6, S7, S8, S9, S10, S11, S12, and S13). Where necessary, structural superpositions for several members of the same family were constructed (Figure 3, Figure 4A). Our results showed very small deviations in any of the structural comparisons analyzed (>1.3 Å). However, the greatest structural differences were located in the oligomerization region of the ALDHs (Figure 4A,Figures S1A, S2A, S3A, S4A, S5A, S6A, S7A, S8A, S9A, S10A, S11A, S12A, and S13A), but the global topology was quite similar among members of the same family. Based on the catalytic domain, the oligomerization domain and the NAD(P)^+^ domain [30], we found that OsALDH12B1 and both members of family 18 were the most divergent from the other rice ALDHs (Figure 3).

In particular, the oligomerization domain (C-terminus) of the ALDHs was the most variable in all models (length, number, curvature angle and folding of secondary structures) (Figure 3, Figure 4A, Figures S1A, S2A, S3A, S4A, S5A, S6A, S7A, S8A, S9A, S10A, S11A, S12A, and S13A). Moreover, we found the largest differences in the angles of curvature of the N-terminal helix in the catalytic domain of OsALDH2B1 and 2B5 (Figure 3), which is projected outward from the general structure. On the other hand, OsALDH22A1 exhibited a longer helix that is folded over itself (Figure 3, Figure S13A). The oligomerization domain of OsALDH12A1 displayed a long loop (Figure 3, Figure S9A).

Protein residues that are linked to biological processes such as protein-protein and protein-ligand interactions are most likely solvent accessible, whereas the residues implicated in protein structure and folding stability are located in the core of the protein. An analysis of structural residue conservation revealed similar residue patterns in all OsALDHs, with the most variable surface residues (depicted in blue) located on the periphery and the conserved residues (depicted in purple) located in the core of the protein structures (Figure 4B, Figures S1B, S2B, S3B, S4B, S5B, S6B, S7B, S8B, S9B, S10B, S11B, S12B, and S13B). The most conserved residues were confined to the catalytic cleft of the rice ALDH structures. OsALDH families 6 and 11 displayed the most conserved catalytic cleft (Figure S4B, Figure S7B), while OsALDH family 2 showed the most variable residue composition in its catalytic cleft (Figure S1B).

The crystal structures of many members of the ALDH superfamily have been shown to exhibit conformational flexibility for the NAD(P) cofactor that reflects a functionally dynamic preference for the oxidized or reduced NAD(P)H/NAD(P)^+^ cofactor. The computational modeling of OsALDH structural surfaces provides insight into the shape of the OsALDH catalytic clefts and enables us to study the important structural features that dictate cofactor specificity (the NAD(P)^+^ binding pocket) within the family (depicted in the lateral views of the structures; Figure 4C, Figures S1C, S2B, S3C, S4C, S5C, S6C, S7C, S8C, S9C, S10C, S11C, S12C, and S13C). The variability of the binding pockets reflects the functional features of the proteins. The ALDHs are known to have variable conformations between non-homologous proteins just like the ligand molecules, but it is also possible that the shapes of different protein binding pockets that bind the same ligand vary [31]. We noticed that the NAD ring was more protected and deeper in the binding pocket of OsALDH class 2, OsALDH3B1, OsALDH3E1, OsALDH3E2, OsALDH6B1, OsALDH7B6, OsALDH12A1 and OsALDH22A1 (Figure 4C, Figures S1C, S5C, S6C, S9C, and S13C), which is similar to previously described NAD-binding patterns for ALDH2 and ALDH3 [30]. However, the NAD ring was less protected in the binding pocket of OsALDH3H1, OsALDH3H2, OsALDH10 and OsALDH11A3 (Figures S3C, S7C, and S8C); and different cofactors were identified for OsALDH5 (β-ME), OsALDH18B1 (glyceraldehyde 3-phosphate) and OsALDH18B2 (adenosine monophosphate). The residue conservation of the binding site and structural comparisons of NADP^+^-dependent ALDHs with known NADP^+^-dependent forms are crucial for predicting the cofactor specificity and the enzymatic mechanism. For example, there is a conserved Glu residue in different positions of the primary sequence that is located on the opposite side of the NAD ring from another conserved Cys residue. These residues have been implicated in proton abstraction from the Cys during the ALDH reaction (Figure 4C, Figures S1C, S2B, S3C, S4C, S5C, S6C, S7C, S8C, S9C, S10C, S11C, S12C, and S13C). We found that both residues were clearly separated from each other by another variable amino acid in ALDH families 2, 6, 10, 11 and 22, and in ALDH3H1, ALDH3H2 and ALDH12A1 (Figures S1C, S2C, S5C, S7C, S8C, and S13C). On the other hand, no separation was found between these residues in family 7 or in ALDH3B1, ALDH3E1 and ALDH3E2 (Figure 4C, Figure S6C), which could be an important factor that influences the thiol extraction step during catalysis by the different ALDHs.

### Electrostatic surface potentials of the OsALDHs

**Figure 5:**
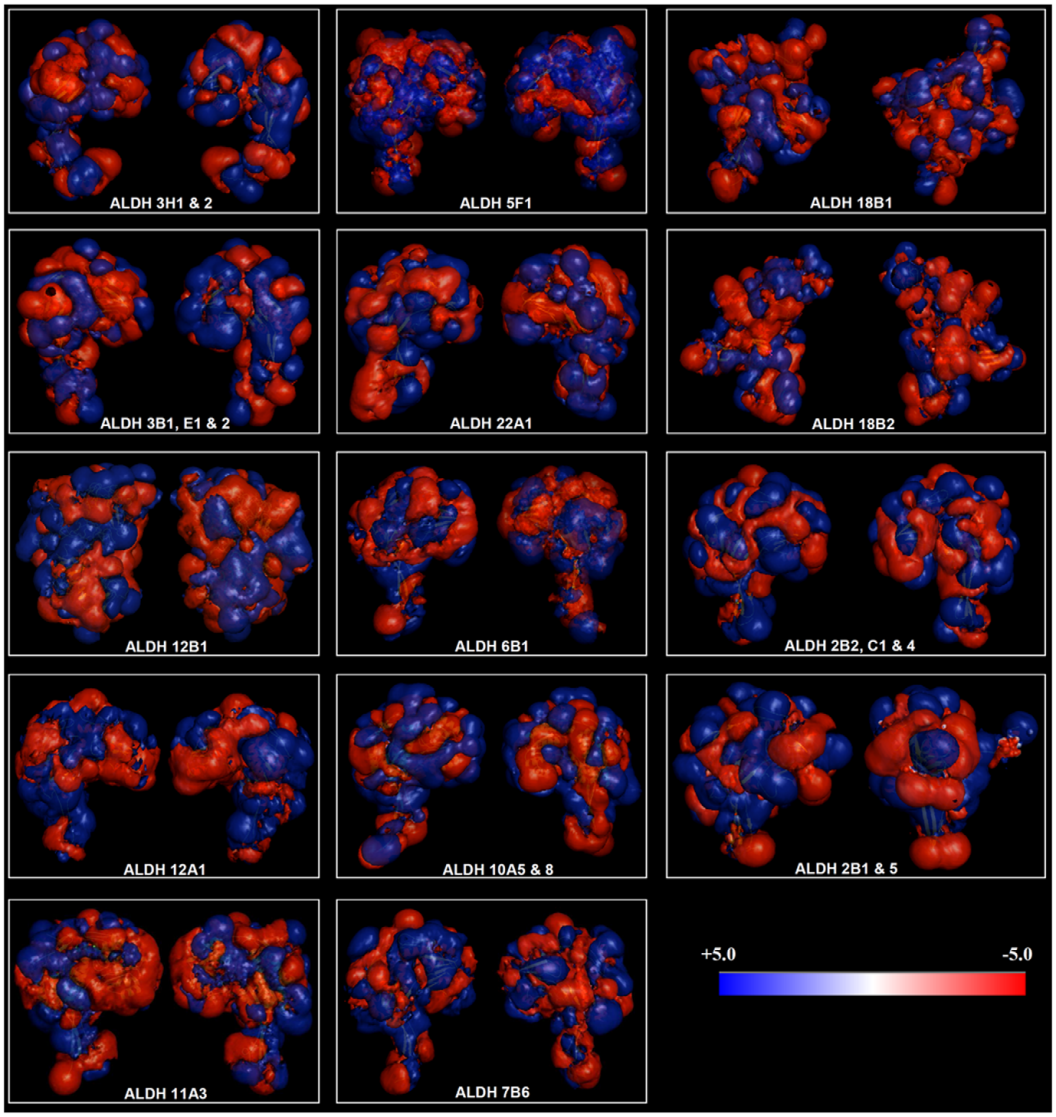
Electrostatic surface of rice ALDH superfamily. Electrostatic potential (isocontour value of ±5 kT/e) surface of all rice ALDHs is depicted in 14 models represent the 21 rice ALDH proteins. In families with more than one member, we considered the isocontour of only one model that has the smaller RMSD value compared to the best theoretical model superimposed.

The Adaptive Poisson-Boltzmann Solver (APBS) package [32] was used to generate the electrostatic surface potentials for all the 21 members of the rice ALDH superfamily, as shown in Figure 4D and the supporting data (Figures S1D, S2D, S3D, S4D, S5D, S6D, S7D, S8D, S9D, S10D, S11D, S12D, and S13D). We examined the charge distribution and patches that differentiate the families and/or family members. The colors in the models depict the different surface properties, with red representing negative charge, blue positive and white neutral (Figure 4D, Figures S1D, S2D, S3D, S4D, S5D, S6D, S7D, S8D, S9D, S10D, S11D, S12D, and S13D). Each protein is represented by six surface plots/views, which correspond to rotations around the vertical (Z) axis (lateral views; front and back views) and the horizontal (X) axis (top and bottom views). Although the overall topologies of these proteins are similar (except for ALDH12B1 and members of family 18), several differences can still be observed. A specific electrostatic potential distribution pattern of the oligomerization domain surface was observed for families 3, 6, and 7 and ALDH12A1. On the other hand, there were clear differences between families (bottom view) as depicted in the isocontour representation data (Figure 5), e.g., families 6 and 11. These charge distribution patterns (Figure 5) (isocontour ranging from −5 kT to −5 kT) could correlate with their different activity properties. In addition, the distribution of these charges denotes differences in the mechanism of action and/or interaction with other proteins and intracellular localization. The surfaces of the catalytic domain and the cofactor-binding domain (top and lateral views) contained the most profound differences in charge distributions. However, the largest positively charged surface included the polymerization region, which spanned the cofactor binding domain, as observed in ALDH families 6, 7 and 10 (Figures S5D, S6D, and 7D) and to a lesser degree in the other families.

## Discussion

Active ALDHs represent an important mechanism for detoxification of reactive aldehyde molecules generated in various developmental growth processes and under environmental stress conditions [5]. The number of identified ALDH genes has increased as more organisms' genomes have been fully sequenced. Here we identified and characterized all *ALDH* genes of rice based upon the standardized *ALDH* gene nomenclature system developed by AGNC [2]. The rice genome contains a total of 21 genes that encode members of ten ALDH families (Table 1). Two (family 2 with 5 genes and family 3 with 5 genes) out of the ten families had more ALDH genes than the other ALDH families. These two classes represent about 50% of the ALDH genes; and family 18 (OsALDH18B1 and OsALDH18B2) that encode the P5CS enzyme was only found in rice (Figure 1, Table 2). A similar observation has been reported for moss ALDHs [21]; two (families 3 and 11) out of the ten moss ALDH families also represent 50% (10 gene members out of the 20 *ALDH* genes) of the moss ALDHs, and family 23 (PpALDH23A1) was solely found in moss [21]. Interestingly, the more abundant plant *ALDH* gene families (families 2, 3, and 11) were not only highly divergent from each other but were also located at the most distant portions in three out of the four clades of the phylogenetic tree (Figure 1). This pattern implies that functional constraints have somehow evolved over time, which might be responsible for the rapid evolution and sequence divergence of these ALDH genes. In addition, the abundance of these genes can be attributed to the diverse environmental conditions to which these plants have been subjected over several million years and the wide variety of substrates they utilize for nutrition. In rice, OsALDH2B2 is responsible for the efficient detoxification of acetaldehydes during re-aeration after submergence, suggesting that class 2 ALDHs play a key role in plant ethanol fermentation [14]. Moreover, different members of the same class (family 2 ALDHs) might be required for different fermentation pathways, justifying the increase in the number of members of the family 2 ALDHs in rice. The members of family 2 are known to require non-identical substrates and do not accumulate in the same tissue at the same time [33]. The same interpretation holds for class 3 OsALDHs (another abundant ALDH family members in rice), which prefer highly variable substrates such as aliphatic and aromatic aldehydes [18]. Class 3 ALDHs play crucial roles in the plant response to abiotic stresses (drought and salt) [5]. The first plant ALDH3 gene, *CpALDH*, was isolated from the resurrection plant *Craterostigma plantagineum* in an attempt to identify genes that help this plant cope with extreme desiccation [15]. Orthologs of this class (family 3) have been identified and characterized in rice (Table 1) and Arabidopsis [5]. In *C. plantagineum*, a resurrection plant that can withstand almost 100% water loss for several years, the activity of CpALDH (ALDH3) was elevated during extreme desiccation to allow the plant to survive in environmental stress conditions in which other plants cannot survive [15]. In addition, over-expression of the *CpALDH* gene confers tolerance to drought and salt stress in transgenic *A. thaliana* [34], and knockout mutations of selected members of class 3 *ALDH* genes are associated with abiotic stress sensitivity [5], [34], indicating that the ALDH gene superfamily can be used as genetic tools to engineer transgenic plants with enhanced environmental stress tolerance. ALDHs are widely distributed in all organisms and are essential for the metabolism (oxidation) of numerous toxic aldehydes into their respective carboxylic acids. These aldehydes are generated from endogenous sources (e.g., de-amination), diet (e.g., ethanol) or pollution (e.g., volatile aldehydes from combustion) [8], [35]. Although the major function of ALDHs is the NAD(P)^+^-dependent oxidation of aldehydes, these enzymes appear to possess multiple catalytic and non-catalytic properties [36]. ALDHs may also play a critical role in cellular homeostasis by maintaining the cellular redox balance; for example, ALDHs may scavenge hydroxyl radicals via the thiol groups of their Cys and Met residues [37]. In addition, ALDH isozymes may contribute to the cellular antioxidant capacity by generating NAD(P)H, which is critical for the regeneration of GSH and may also function as a direct antioxidant [38]. A comparative study of the entire members of the ALDH protein superfamily at the structural level has not been performed before. Here we used computational modeling to report the 3D structural features of members of the entire rice ALDH protein superfamily and to highlight specific structural properties and functional implications of the NAD(P)^+^ binding cleft within the members of the same or different families.

Although protein sequence alignment of the members of the ALDH superfamily reveals identities of less than 40%, these proteins do share a common overall folding pattern with discernable domains in each monomeric subunit. Domain organization is an intrinsic element of protein structure. The majority of these proteins have distinct catalytic, cofactor-binding and oligomerization domains that can act independently or cooperatively to achieve a unique function [39]. ALDHs have multiple catalytic and non-catalytic functions in addition to their roles in aldehyde metabolism [19].

The oligomerization domain of rice ALDHs is the most variable domain. We distinguished four different groups based on this domain: group 1 (families 2, 5, 6, 7, 10 and 11) is characterized by two β-sheets and a short α-helix; group 2 (families 3 and 22) is characterized by two β-sheets and a long α-helix; group 3 (ALDH12A1) is characterized by the integration of the C-terminal domain into the catalytic domain so that the oligomerization domain is characterized by a long loop; and group 4 (ALDH12B1 and the family 18) has structures and topologies that are different from the rest. The C-terminal domain of ALDHs is implicated in the oligomerization state of the proteins *in vivo* [40]. In general, these tails determine the binary and quaternary structure of the protein. Due to the diversity of this domain, we speculate that different families might be thermodynamically more stable in different polymerization states. The thermodynamic stability of the protein subsequently influences the catalytic state and enzymatic properties of the protein. The C-terminal tail is not the only factor that influences the formation of dimers or tetramers. The interactions between amino acids (mainly in the C-terminal region) as well as interactions with other domains of the protein (e.g., the catalytic domain) might influence the maintenance of stable dimers or oligomers [40]. The oligomerization state of ALDHs is also important for catalytic function, which has been previously demonstrated for betaine dehydrogenase (ALDH10) [41]. The catalytic pocket entrance is well conserved in all rice ALDHs except in families 2 and 10 (Figures S1, S2, and S7), which have two variable amino acids in the catalytic entrance that are close to the cofactor. These variable amino acids might partially affect the anatomy of the cavity that binds the cofactor. In addition, the accessibility and ability of the enzyme to react with specific substrates might also be affected.

There are specific amino acids (Cys and Glu) that are crucial for substrate specificity and catalytic activity at the molecular level. In NAD(P)^+^-dependent ALDH reactions, the substrate enters the catalytic site through the cavity. An interaction between the cofactor and the enzyme (via the Rossmann fold) allows the enzyme to isomerize after reduction of the cofactor. The Cys residue in the “attacking” conformation [42] carries out a nucleophilic attack on the carbonyl carbon of the aldehyde substrate to form a thiohemiacetal intermediate [43]. The Glu residue helps a water molecule in the active site to make a nucleophilic attack on the carbonyl carbon, abstracting the sulfur group. Interestingly, both amino acids (Cys and Glu) are conserved in most OsALDHs, but their predicted positions in the primary structures are different. Many other residues that comprise the catalytic pocket interact with NAD(P)^+^ to hold it in place. These residues are variable depending on the ALDH family; some of them are conserved and crucial for efficient catalysis [44], while others have key roles in protein folding [45].

We examined the binding mode of the adenosine moiety of the nucleotides in rice ALDHs and found it to be conserved across taxa. Unlike the interaction between the NADP(H) phosphate group and the ALDH residues, this interaction involves in the formation of hydrogen bonds between the enzyme residues and the hydroxyl groups of the adenosine ribose [46]. In the case of ALDHs that bind NAD^+^ better than NADP^+^, there is a negatively charged amino acid residue that interacts with the adenosine ribose. Whereas, this residue is uncharged in ALDHs that preferentially bind NAD(P)H [47]. Similarly, the ALDHs that bind NADP^+^ with higher affinity than NAD^+^ have an uncharged residue at a position equivalent to E195 [48]. In addition, enzymes that prefer NAD(P)^+^ have an arginine residue near E195 [42]. This interaction with the phosphate group of NADP^+^ allows the enzyme to switch between the NAD^+^- and the NADP^+^-bound conformations. We classified rice ALDHs based on their NAD(P)^+^ binding preferences as defined by the enzymatic residues close to the ribose phosphate. Proteins in which E195 and adjacent residues are substituted with uncharged amino acids (A, V, L, I, T, and C) comprise the first group and include ALDH2B1, ALDH2B5, ALDH12A1, and members of families 5, 6, 7, and 10. This residue substitution corresponds to an enzyme that prefers NADP^+^. Group 2 ALDHs (ALDH2B2, ALDH2C1, ALDH2C4, ALDH3B1, ALDH3E1, ALDH3E2 and members of family 11) have a negatively charged amino acid (E or K) at or near E195, hence prefer NAD^+^ as a cofactor. In general, substituting crucial amino acids involving in the NAD(P)^+^ cofactor binding into polar or charged amino acids will result in changing the enzyme cofactor specificity from NADP+ to NAD+ [49], [50]. The third group (ALDH3H1 and ALDH3H3) contains an arginine (R) residue at position 195 and possibly switches between NAD^+^ and NADP^+^ cofactors.

The interaction between the ALDHs and the nicotinamide moiety is poorly characterized because there are few crystallized structures that contain NAD^+^ [42]. The nicotinamide ring in the active site of the ALDHs is dynamic, hence impedes crystallization of the complex. However, this movement might be essential for the correct positioning of the catalytic residues and the hydrolytic water during the course of the ALDH-cofactor reaction [38].

The macromolecular interaction between proteins provides key information for elucidating their biological function [43], [51]. Although different proteins in a molecular network are independent, they should not be considered as isolated components because they are molecularly arranged in networks in the biochemical pathways. The electrostatic potential of an enzyme is another key feature related to substrate specificity and catalytic turnover. Differences in the electrostatic potential at or near the surface of isofunctional enzymes may correlate with different binding partners or adaptations to tissue-specific environmental conditions. Comparative analyses of protein electrostatic potentials and structural modeling are key tools for enzyme classification and characterization. The analysis of the electrostatic potentials of rice ALDH enzymes belonging to different families has allowed us to organize them and compare their possible functional differences. Moreover, we identified specific protein surface interaction properties (protein-protein, protein-cofactor and/or protein-substrate interactions) in different domains of the ALDHs. In a protein structure-based molecular analysis, the challenge is to relate the differences observed in protein structures to differences in enzyme activity. The molecular electrostatic potential is an important informative property for studying enzymatic function and interaction [52]. It has been previously demonstrated that the electrostatic potential pattern of an enzyme is one of key determinants for its functional conservation [53], [54], [55]. Here we identified a subtle but evident pattern in the surface electrostatic potentials of members of the rice ALDH superfamily. The distribution of positive charges was the same in all of the ALDH C-terminal domains. These domains are crucial for dimerization and oligomerization, indicating that oligomerization occurs in a similar manner within the ALDH protein families. However, the electrostatic charge distribution of the cofactor-interacting domain varies from one enzyme to another, which reflects differences in cofactor affinity and specificity. In the topology of the ALDH families, the most variable domains (in terms of the electrostatic potential) seem to be the catalytic and cofactor domains. In rice ALDH families 3, 6, 7 and 10, these domains predominantly have positive charges, but the opposite was observed for ALDH families 2, 8, 11, 12 and 22. This differential distribution could directly affect the interaction of the protein with other partners and target it to a different sub-cellular localization. Using computational modeling, we predicted for the first time the intrinsic the structural conformations and features of each ALDH enzyme involved in biological pathways. To derive relationships between enzyme kinetics and molecular interactions, between enzymes and substrates or other critical constituents of biochemical pathways, it is necessary to understand the enzyme's structure and the molecular properties of its functional domains in detail. Comparisons of 3D structural properties provide information that is complementary to genomic sequences. Our comparison provided insight into the structural and functional features of the rice ALDH protein superfamily and identified some novel properties of these important enzymes.

## Materials and Methods

### The rice ALDH database search, revised gene annotation and phylogenetic analysis

The ALDH protein sequences of *Arabidopsis thaliana* [18], Pfam 00171 (ALDH family) protein domains (http://pfam.sanger.ac.uk/), PS00070 (ALDH cysteine active site), PS00687 (ALDH glutamic acid active site), KOG2450 (aldehyde dehydrogenase), KOG2451 (aldehyde dehydrogenase), KOG2453 (aldehyde dehydrogenase) and KOG2456 (aldehyde dehydrogenase) were used as queries to search the rice genomic database (TIGR Rice Annotation Release 4, http://tigrblast.tigr.org/eukblast/index.cgi?project=osa1) to identify ALDH and ALDH-like sequences using BLASTX, BLASTN and BLAST (low complexity filter, Blosum62 substitution matrix) [56]. All sequences with an E-value of ℜ1e-6 were selected for manual inspection. Protein motifs were additionally queried using the Pfam, PROSITE, CDD (Conserved Domain Database) or CDART (Conserved Domain Architecture Retrieval Tool) tools [57], [58]. The deduced rice ALDH polypeptides were analyzed using tools available at the ExPASy Proteomics Server (http://www.expasy.ch/tools/). The deduced ALDH polypeptides were annotated using the criteria established by the ALDH Gene Nomenclature Committee (AGNC) [2]. The AGNC nomenclature has been applied to the annotation of several eukaryotic genomes, including *A. thaliana* [18]. Deduced amino acid sequences that were more than 40% identical to other previously identified ALDH sequences composed a family, and sequences with more than 60% identity composed a protein subfamily. Deduced amino acid sequences with less than 40% identity described a new ALDH protein family.

For the phylogenetic analysis, the *A. thaliana* (The Arabidopsis Information Resource, TAIR; http://www.arabidopsis.org/), *Physcomitrella patens* ssp. Patens, and *Chlamydomonas reinhardtii* (Genome Resources of the US Department of Energy Joint Genome Institute; http://genome.jgi-psf.org/) ALDH superfamilies were retrieved and used together with the rice ALDH superfamily to generate a phylogenetic tree using ClustalW [59]. The alignments were created using the Gonnet protein weight matrix, multiple alignment gap opening/extension penalties of 10/0.5 and pairwise gap opening/extension penalties of 10/0.1. These alignments were adjusted using Bioedit V 7.0.5.3 [60]. Portions of sequences that could not be reliably aligned were eliminated. Phylogenetic trees were generated by the neighbor-joining method (NJ), and the branches were tested with 1,000 bootstrap replicates. Both trees were visualized using Treedyn [61].

### Transposon annotation in rice *ALDH* genes

DNA sequences of the rice ALDH genes were downloaded from the Rice Genome Annotation Project website (http://rice.plantbiology.msu.edu) and used for transposon annotation. The rice repeat database (unpublished, Dr. Ning Jiang, Michigan State University) was chosen to screen the rice genes using the RepeatMasker software with default parameters (http://www.repeatmasker.org). Subsequently, reads obtained by RepeatMasker were checked manually to determine target site duplications (TSDs) and terminal repeats of transposons.

### Protein structural modeling and conservational analysis of the rice ALDH superfamily

To understand the structural and molecular conformational differences between the members of the rice ALDH protein superfamily as well as their protein-protein interaction characteristics and ligand-protein interaction properties, the 21 deduced ALDH protein sequences were modeled using the top ten PDB closed template structures by I-Tasser [62]. An initial structural model was generated for each ALDH and subjected to energy minimization with GROMOS96 [63] implemented in DeepView/Swiss-PDBViewer v3.7 [64] to improve the van der Waals contacts and correct the stereochemistry of the model. For each sequence analyzed, the quality of the model was assessed by checking the protein sterology with PROCHECK [65] and the protein energy with ANOLEA [66]. Ramachandran plot statistics for the models were calculated to show the number of protein residues in the favored regions.

The binding site for each ALDH structure was predicted based on analogs with similar binding sites and BS-scores. The structural models were also predicted based on the TM-score (the scale for measuring the structural similarity between two structures), IDEN (percentage of sequence identity in the structurally aligned region), the coverage of the alignment by TM-align, the COV of the model, and the structural alignment (which is equal to the number of structurally aligned residues divided by the length), with a BS-score of >0.5 signifying a binding site predicted with high confidence. The ligands in the analog structure were then transferred to the model, and the fitness of the ligand-model complex (BS-score) was calculated by comparing the local structure and sequence similarity in the binding site region.

To identify functional regions of known three dimensional protein structures, ConSurf conservation analysis [67] was used to estimate the evolutionary conservation score of the residues, which is the degree of conservation of the amino acid in 50 close homologs (identification of functional regions in proteins by surface-mapping of the phylogenetic information).

Electrostatic Poisson-Boltzmann (PB) potentials were obtained using APBS [32] molecular modeling software in PyMol 0.99 (DeLano Scientific LLC) with AMBER99 [68] to assign the charges and radii to all of the atoms (including hydrogens), which were added and optimized with PDB2PQR [69], a Python software package that automates many of the common tasks used to prepare structures for continuum electrostatics calculations and provides a platform-independent tool for converting protein files in the PDB format to the PQR format. Fine grid spaces of 0.35 Å were used to solve the linearized PB equation in sequential-focusing multigrid calculations in a mesh of 130 points per dimension at 310.00 K. The dielectric constants were 2 for the protein and 80.00 for water. The output mesh was processed in the scalar OpenDX format to render isocontours and maps onto the surfaces with PyMOL 0.99. Potential values are given in units of kT per unit charge (k, Boltzmann's constant; T, temperature).

## Supporting Information

Table S1 Transposable elements nested on the rice ALDH gene superfamily.
(0.05 MB DOC)

Table S2 Structural-dependent modeling parameters for the rice ALDH protein superfamily.
(0.06 MB DOC)

Figure S1 Detail structural conformation and conservation analysis of selected members of rice ALDH family 2, OsALDH2B2, 2C1 and 2C4. (A) General structure (cartoon diagram) of indicated members of family 2 ALDH showing the superimposition of OsALDH2B2 (red), 2C1 (blue) and 2C4 (turquoise) with RMSD calculated for each superimposition. Represented structures were rotated at 180°. (B) Best predicted ALDH2C4 model (2D-structure) was subject to consurf-conservational analysis searching for close homologous sequences with known structures using PSI-BLAST. The protein was finally visualized using FirstGlance in Jmol with the conservation scores colour-coded. The conserved and variable residues are presented as a space-filled model and coloured according to the conservation scores. A detailed view of the cavity holding up the NAD(P)^+^ cofactor (stick model and van der Walls spheres) is shown in high magnification. (C) Surface conformation of ALDH2C4 (lateral views represent 180° rotation) showing the secondary structure elements inside. The morphology of the cavity accommodating NAD(P)^+^ cofactor is represented in high magnification. Detailed organization of the predicted amino acids of the pocket is represented in blue. Space-filled representation of van der Waals surface of the cofactor, and the catalytic residues (Cys 303 in green and Glu 269 in red) are opposite positioned. (D) Electrostatic surface potential showing different views of ALDH2C4 structure. The surface colours are clamped at red (−1) or blue (+1). Top and bottom views are highlighted with a white line coming from front view.
(8.51 MB TIF)

Figure S2 Detail structural conformation and conservation analysis of selected members of rice ALDH family 2, OsALDH2B1 and OsALDH2B5. (A) General structure (cartoon diagram) of the superimposition of OsALDH2B1 (light pink) and 2B5 (yellow) with RMSD calculated for each superimposition is shown. Represented structures were rotated at 90°. (B) Best predicted ALDH2B5 model (2D-structure) was subjected to consurf conservational analysis searching for close homologous sequences with known protein structures using PSI-BLAST. The protein was finally visualized using FirstGlance in Jmol, with the conservation scores colour-coded onto its surface. The conserved and variable residues are presented as a space-filled model, and coloured according to the conservation scores. A detailed view of the cavity holding up the NAD(P)^+^ cofactor (stick model and van der Walls spheres) is shown in high magnification. (C) Surface conformation of ALDH2B5 lateral views (rotated 180°), showing the secondary structure elements inside is depicted. The morphology of the cavity accommodating the cofactor is represented in high magnification. Detailed organization of the amino acid (aa) residues of the pocket is represented in blue. Stick model of the cofactor, and the catalytic aa residues (Cys 354 [green] and Glu 320 [red]), at opposite positions are shown. (D) Electrostatic surface potential showing all possible views of the ALDH2B5 structure. The surface colours are clamped at red (−1) or blue (+1). Top and bottom views are highlighted with a white line coming from the front view.
(8.13 MB TIF)

Figure S3 Detail structural conformation and conservation analysis of selected members of rice ALDH family 3, OsALDH3H1 and 3H2. (A) General structure (cartoon diagram) of the superimposition of OsALDH3H1 (green) and 3H1 (blue) with RMSD calculated for each superimposition is shown. Represented structures were rotated at 180°. (B) The best predicted ALDH3H2 model (2D-structure) was subjected to consurf conservational analysis searching for close homologous sequences of the protein of known structures using PSI-BLAST. The protein was visualized using FirstGlance in Jmol, with colour-coded conservation scores of its surface. The variable and conserved residues are presented as a space-filled model, and coloured according to the conservation scores. A detailed view of the cavity holding up the NAD(P)^+^ cofactor (stick model and van der Walls spheres) is shown. (C) Surface conformation of the ALDH3H2 lateral views (rotated 180°) is depicted showing the secondary structure elements inside. The morphology of the cavity accommodating the cofactor is represented in high magnification. Detail view organization of the predicted amino acids (aa) of the pocket is represented in blue colour. Space-filled representation of van der Waals surface of the cofactor, and the catalytic opposite positioned aa Cys 247 (green) and Glu 341 (red) is shown. (D) Electrostatic surface potential showing all the possible views of the ALDH3H2 structure. The surface colours are clamped at red (−1) or blue (+1). Top and bottom views are highlighted with a white line coming from the front view.
(8.50 MB TIF)

Figure S4 Detail structural conformation and conservation analysis of rice ALDH family 5 (ALDH5F1). (A, B, D) structural descriptions are similar to that of Figure S3 with exception of superimposition. (C) Detail view organization of the predicted amino acids (aa), which are close to the chemical ligand β-mercaptoethanol (β-ME) and the NAD(P)^+^ cofactor is represented in blue colour. Space-filled representation of van der Waals surface of β-ME, and the interacting aa Cys 332, VAL333, THR331, PHE201 and LEU208 are shown in green colour.
(8.45 MB TIF)

Figure S5 Detail structural conformation and conservation analysis of rice ALDH family 6 (ALDH6B1). (A, B, D) Detail description similar to that of Figure S3 with the exception of superimposition. The secondary structure elements are depicted in different colours: α-helix (red), β-sheet (yellow) and coils (green). (C) Space-filled representation of van der Waals surface of the cofactor, and the catalytic opposite positioned amino acids Cys 318 (green) and Glu 418 (red) are here shown.
(8.55 MB TIF)

Figure S6 Detail structural conformation and conservation analysis of rice ALDH family 7 (ALDH7B6). (A, B, D) The structural description is similar to that of Figure S3 with the exception of superimposition. The secondary structure elements (A) are shown in different colours: α-helix (red), β-sheet (yellow) and coils (green). (C) The space-filled representation of van der Waals surface of the cofactor, and the catalytic opposite positioned amino acids Cys 301 (green) and Glu 397 (red) are shown.
(8.42 MB TIF)

Figure S7 Detail structural conformation and conservation analysis of rice ALDH family 10 (ALDH10A5 and 10A8). (A, B, C) The structural description is similar to that of Figure S3. For the superimposition (A), OsALDH10A5 is represented in yellow and OsALDH10A8 is in blue. (C) The space-filled representation of van der Waals surface of the cofactor, and the catalytic opposite positioned amino acids Cys 247 (green) and Glu 395 (red) are here depicted.
(8.26 MB TIF)

Figure S8 Detail structural conformation and conservation analysis of rice ALDH family 11 (ALDH11A3). (A, B, D) The structural description is similar to that of Figure S3 with the exception of superimposition. The secondary structure elements (A) are shown in different colours: α-helix (red), β-sheet (yellow) and coils (green). (C) The space-filled representation of van der Waals surface of the cofactor, and the catalytic opposite positioned amino acids Cys 301 (green) and Glu 394 (red) are shown.
(9.04 MB TIF)

Figure S9 Detail structural conformation and conservation analysis of rice ALDH family 12 (ALDH12A1). (A, B, D) The structural description is similar to that of Figure S3 with the exception of superimposition. The secondary structure elements (A) are shown in different colours: α-helix (red), β-sheet (yellow) and coils (green). (C) The space-filled representation of van der Waals surface of the cofactor, and the catalytic opposite positioned amino acids Cys 331 (green) and Glu 435 (red) are shown.
(8.15 MB TIF)

Figure S10 Detail structural conformation and conservation analysis of rice ALDH family 12 (ALDH12B1). (A, B, D) The structural description is similar to that of Figure S3 with the exception of superimposition. The secondary structure elements (A) are shown in different colours: α-helix (red), β-sheet (yellow) and coils (green). (C) The space-filled representation of van der Waals surface (in green colour) is represented, and the predicted amino acids comprising the pocket, which accommodate the NAD(P)+ cofactor F499, T500, R501, T502 and V505, are shown.
(7.77 MB TIF)

Figure S11 Detail structural conformation and conservation analysis of rice ALDH family 18 (ALDH18B1). (A, B, D) The structural description is similar to that of Figure S3 with the exception of superimposition. The secondary structure elements (A) are shown in different colours; α-helix (red), β-sheet (yellow) and coils (green). (B) A detailed view of the cavity holding up the molecule glyceraldehyde 3-Phosphate (stick model and van der Walls spheres) is shown. (C) Detail view organization of the predicted amino acids (aa) close to the chemical ligand glyceraldehyde 3-phosphate and the NAD(P)+ cofactor is depicted in blue colour. The space-filled representation of van der Waals surface of the molecule glyceraldehyde 3-Phosphate, and the interacting aa D658, N544, A542 and R415 are shown in green colour.
(7.98 MB TIF)

Figure S12 Detail structural conformation and conservation analysis of rice ALDH family 18 (ALDH18B2). (A, B, D) The structural description is similar to that of Figure S3 with the exception of superimposition. The secondary structure elements (A) are shown in different colours: α-helix (red), β-sheet (yellow) and coils (green). (C) Detail view organization of the predicted amino acids (aa) close to close to the chemical ligand adenosine monophosphate and the NAD(P)^+^ cofactor is shown in blue colour. Space-filled representation of van der Waals surface of the molecule adenosine monophosphate, and the interacting aa S403, T421, C422 and L706 are shown in green colour.
(7.95 MB TIF)

Figure S13 Detail structural conformation and conservation analysis of rice ALDH family 22 (ALDH22A1). (A, B, D) The structural description is similar to that of Figure S3 with the exception of superimposition. The secondary structure elements (A) are shown in different colours: α-helix (red), α-sheet (yellow) and coils (green). (C) The space-filled representation of van der Waals surface of the cofactor, and the catalytic opposite positioned amino acids Cys 332 (green) and Glu 433 (red) are shown.
(8.99 MB TIF)
