# Supplementary material for: Modeling-Dependent Protein Characterization of the Rice Aldehyde Dehydrogenase (ALDH) Superfamily Reveals Distinct Functional and Structural Features
Source: PLoS One. 2010 Jul 12;5(7):e11516. doi: 10.1371/journal.pone.0011516 (PMC2902511; doi:10.1371/journal.pone.0011516)
Supplement: Table S1 — Transposable elements nested on the rice ALDH gene superfamily. (0.05 MB DOC) [file pone.0011516.s001.doc]

**Supplemental Table S1:**

| **ALDH family** | **Gene annotation** | **Gene locus** | **Name of transposon** | **Superfamily** | **Location** | **Orientation** | **Transposon region** | **5' TSD** | **3' TSD** | **Genic region** |
| --- | --- | --- | --- | --- | --- | --- | --- | --- | --- | --- |
| Family 2 | OsALDH2C4 | Os01g40860 | Gaijin | MITE/Tourist | 3279-3425 | - | 1-147 | AGA | AGA | 1^st^ intron |
| Family2 | OsALDH2C1 | Os01g40870 | OS24 | MITE/Stow | 2618-2797 | - | 55-241 | TA |  | 1^st^ intron |
|  |  |  | OS6 | MITE/Stow | 2797-3043 | - | 1-255 | TT | TA | 1^st^ intron |
|  |  |  | OS24 | MITE/Stow | 3044-3091 | - | 1-48 |  | TA | 1^st^ intron |
| Family 18 | OsALDH18B2 | Os01g62900 | IO2744 | DNAnona/Helitron | 2509-5085 | - | 1-2577 |  |  | 6^st^ intron |
|  |  |  | OS1 | MITE/Stow | 7348-7497 | + | 1-157 | TA | TA | 15^st^ intron |
| Family 3 | OsALDH3E1 | Os02g43194 | OS6 | MITE/Stow | 1571-1824 | - | 1-255 | TA | TA | 3^rd^ intron |
| Family 3 | OsALDH3E2 | Os02g43280 | OS9 | MITE/Stow | 1305-1536 | - | 1-240 | TA | TA | 3^rd^ intron |
|  |  |  | Ditto | MITE/Tourist | 2022-2273 | + | 1-244 | TAA | TAA | 3^rd^ intron |
|  |  |  | OS8 | MITE/Stow | 2374-2633 | - | 1-257 | TA | TA | 3^rd^ intron |
|  |  |  | OS6 | MITE/Stow | 3291-3550 | + | 1-260 | TA | TA | 3^rd^ intron |
| Family 3 | OsALDH3B1 | Os04g45720 | OS9 | MITE/Stow | 2482-2719 | - | 1-240 | TA | TA | 3^rd^ intron |
| Family 6 | OsALDH6B1 | Os07g09060 | OS8 | MITE/Stow | 4842-5095 | + | 1-257 | TA | TA | 14^st^ intron |
| Family 7 | OsALDH7B6 | Os09g26880 | Os0089 | DNAnona/MULE | 6068-6808 | - | 1-743 | ATTAGCAAA | ATTAGCAAA | 13^rd^ intron |
|  |  |  | Os0299 | MITE/Tourist | 6907-7057 | + | 1-51 | TAA |  | 13^rd^ intron |
|  |  |  | OS6 | MITE/Tourist | 7058-7389 | + | 1-332 | TAA | TAA | 13^rd^ intron |
|  |  |  | Os0299 | MITE/Tourist | 7390-7442 | + | 50-102 |  | TAA | 13^rd^ intron |
| Family 3 | OsALDH3H1 | Os12g07810 | Os3327 | MITE/Stow | 4891-5126 | + | 1-233 | TA | TA | 3^rd^ intron |

Note: Os24 within Os01g40870 and Os0299 in Os09g26880 are nested by OS6s, respectively.
