# Supplementary material for: Modeling-Dependent Protein Characterization of the Rice Aldehyde Dehydrogenase (ALDH) Superfamily Reveals Distinct Functional and Structural Features
Source: PLoS One. 2010 Jul 12;5(7):e11516. doi: 10.1371/journal.pone.0011516 (PMC2902511; doi:10.1371/journal.pone.0011516)
Supplement: Table S2 — Structural-dependent modeling parameters for the rice ALDH protein superfamily. (0.06 MB DOC) [file pone.0011516.s002.doc]

| **Gene locus** | **Gene annotation** | **C-score** | **TM-Score** | **Nº of decoys** | **Cluster density** | **RMSD (Å)** | **Template (higher Z-score)** | **% Identity with the template** | **Norm.**  **Z-score** |
| --- | --- | --- | --- | --- | --- | --- | --- | --- | --- |
| Os06g15990 | ALDH2B1 | -0.60 | 0.64±0.13 | 1978 | 0.1273 | 8.9±4.6 | 1ag8C | 59 | 10.23 |
| Os02g49720 | ALDH2B5 | -1.08 | 0.58±0.14 | 1262 | 0.0773 | 10.1±4.6 | 1ag8A | 59 | 11.22 |
| Os01g40870 | ALDH2C1 | 0.79 | 0.82±0.08 | 2847 | 0.5048 | 5.6±3.5 | 1ag8A | 54 | 10.16 |
| Os02g43194 | ALDH3E1 | -0.47 | 0.65±0.13 | 2075 | 0.1527 | 8.3±4.5 | [1ad3A](http://www.rcsb.org/pdb/explore/explore.do?structureId=1ad3) | 44 | 10.45 |
| Os02g43280 | ALDH3E2 | -0.24 | 0.68±0.12 | 2063 | 0.1987 | 7.8±4.4 | [1ad3A](http://www.rcsb.org/pdb/explore/explore.do?structureId=1ad3) | 41 | 9.21 |
| Os11g08300 | ALDH3H2 | -0.38 | 0.66±0.13 | 2045 | 0.1687 | 8.1±4.4 | [1ad3A](http://www.rcsb.org/pdb/explore/explore.do?structureId=1ad3) | 48 | 9.31 |
| Os12g07810 | ALDH3H1 | 0.34 | 0.76±0.10 | 2124 | 0.3324 | 6.5±3.9 | [1ad3A](http://www.rcsb.org/pdb/explore/explore.do?structureId=1ad3) | 46 | 9.20 |
| Os04g45720 | ALDH3B1 | 0.23 | 0.74±0.11 | 2096 | 0.3133 | 6.8±4.0 | [1ad3A](http://www.rcsb.org/pdb/explore/explore.do?structureId=1ad3) | 42 | 10.60 |
| Os02g07760 | ALDH5F1 | -0.35 | 0.67±0.13 | 1928 | 0.1575 | 8.2±4.5 | [3ek1A](http://www.rcsb.org/pdb/explore/explore.do?structureId=3ek1) | 57 | 5.77 |
| Os07g09060 | ALDH6B1 | 0.53 | 0.78±0.09 | 2294 | 0.3862 | 6.3±3.8 | 1t90A | 42 | 5.56 |
| Os09g26880 | ALDH7B6 | 1.62 | 0.94±0.05 | 3000 | 1.2195 | 4.0±2.7 | 2j6lA | 58 | 5.47 |
| Os08g32870 | ALDH10A8 | 1.70 | 0.95±0.05 | 3000 | 1.2500 | 3.8±2.6 | 3ed6A | 44 | 5.73 |
| Os04g39020 | ALDH10A5 | 1.75 | 0.96±0.05 | 3000 | 1.2500 | 3.7±2.5 | 1ag8A | 42 | 12.90 |
| Os08g34210 | ALDH11A3 | 0.89 | 0.83±0.08 | 2999 | 0.5846 | 5.4±3.4 | 1euhA | 52 | 4.33 |
| Os05g45960 | ALDH12A1 | -0.21 | 0.69±0.12 | 2085 | 0.2294 | 7.6±4.3 | [1a4sA](http://www.rcsb.org/pdb/explore/explore.do?structureId=1a4s) | 19 | 5.34 |
| Os07g48920 | ALDH22A1 | -0.63 | 0.63±0.13 | 1647 | 0.1333 | 9.2±4.6 | [1a4sA](http://www.rcsb.org/pdb/explore/explore.do?structureId=1a4s) | 29 | 8.74 |
| Os01g40860 | ALDH2C4 | 1.20 | 0.88±0.07 | 3000 | 0.7092 | 4.8±3.1 | 1bxsA | 53 | 11.37 |
| Os06g39230 | ALDH2B2 | 1.45 | 0.92±0.06 | 3000 | 0.9346 | 3.9±2.7 | 1bxsA | 54 | 11.37 |
| Os05g38150 | ALDH18B1 | -1.79 | 0.50±0.15 | 138 | 0.0377 | 12.5±4.3 | 2h5gA | 49 | 5.28 |
| Os1g62900 | ALDH18B2 | -2.17 | 0.46±0.15 | 121 | 0.0261 | 13.6±4.0 | 2h5gA | 49 | 5.34 |
| Os12g40440 | ALDH12B1 | -2.26 | 0.45±0.14 | 174 | 0.0852 | 14.1±3.8 | 1uxtA | 14 | 2.63 |

**Supplemental Table S2:**
