# Supplementary figures and images for: Modeling-Dependent Protein Characterization of the Rice Aldehyde Dehydrogenase (ALDH) Superfamily Reveals Distinct Functional and Structural Features
Source: PLoS One. 2010 Jul 12;5(7):e11516. doi: 10.1371/journal.pone.0011516 (PMC2902511; doi:10.1371/journal.pone.0011516)

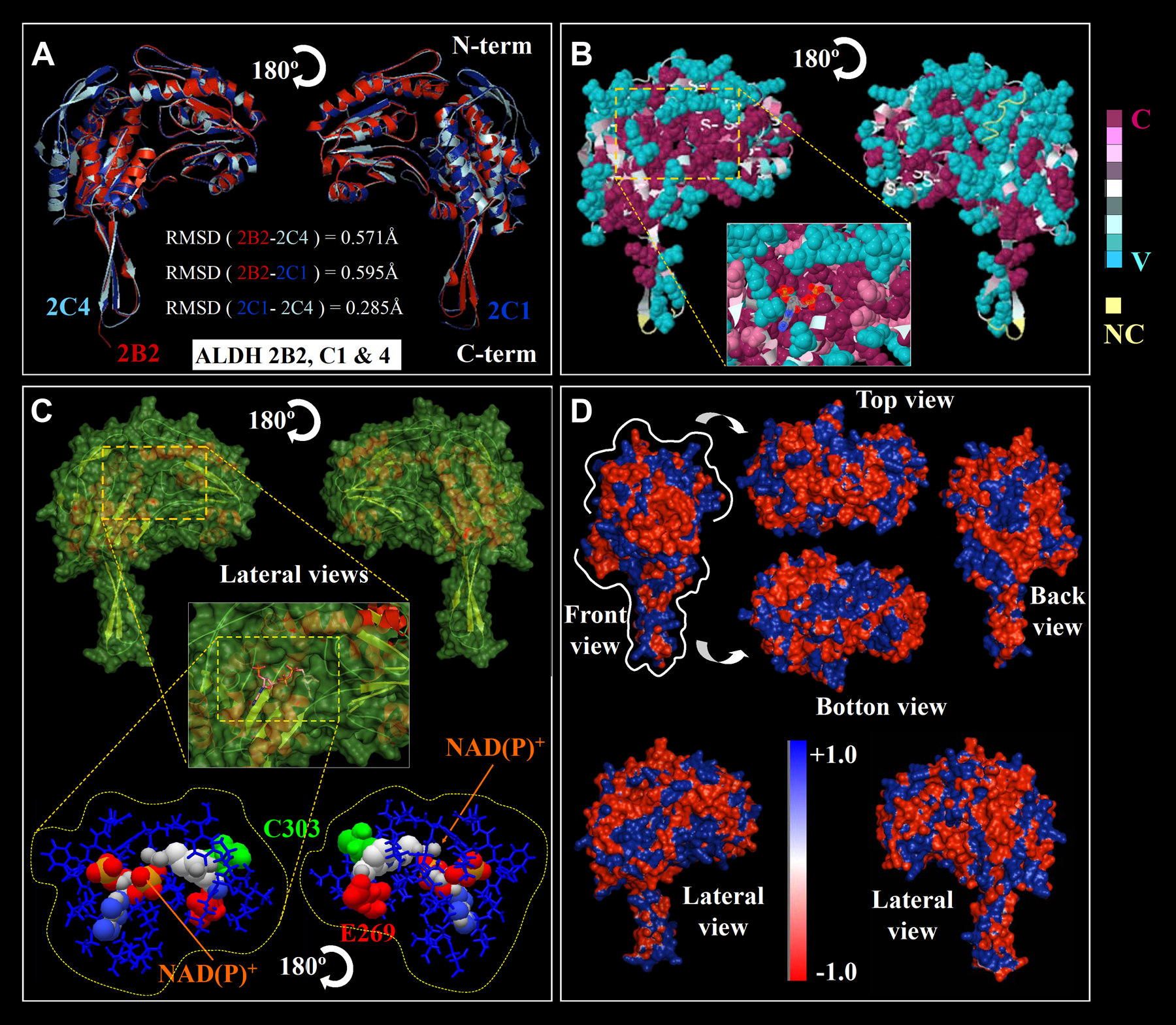

Supplement: Figure S1 — Detail structural conformation and conservation analysis of selected members of rice ALDH family 2, OsALDH2B2, 2C1 and 2C4. (A) General structure (cartoon diagram) of indicated members of family 2 ALDH showing the superimposition of OsALDH2B2 (red), 2C1 (blue) and 2C4 (turquoise) with RMSD calculated for each superimposition. Represented structures were rotated at 180°. (B) Best predicted ALDH2C4 model (2D-structure) was subject to consurf-conservational analysis searching for close homologous sequences with known structures using PSI-BLAST. The protein was finally visualized using FirstGlance in Jmol with the conservation scores colour-coded. The conserved and variable residues are presented as a space-filled model and coloured according to the conservation scores. A detailed view of the cavity holding up the NAD(P)+ cofactor (stick model and van der Walls spheres) is shown in high magnification. (C) Surface conformation of ALDH2C4 (lateral views represent 180° rotation) showing the secondary structure elements inside. The morphology of the cavity accommodating NAD(P)+ cofactor is represented in high magnification. Detailed organization of the predicted amino acids of the pocket is represented in blue. Space-filled representation of van der Waals surface of the cofactor, and the catalytic residues (Cys 303 in green and Glu 269 in red) are opposite positioned. (D) Electrostatic surface potential showing different views of ALDH2C4 structure. The surface colours are clamped at red (−1) or blue (+1). Top and bottom views are highlighted with a white line coming from front view. (8.51 MB TIF) [file pone.0011516.s003.tif]

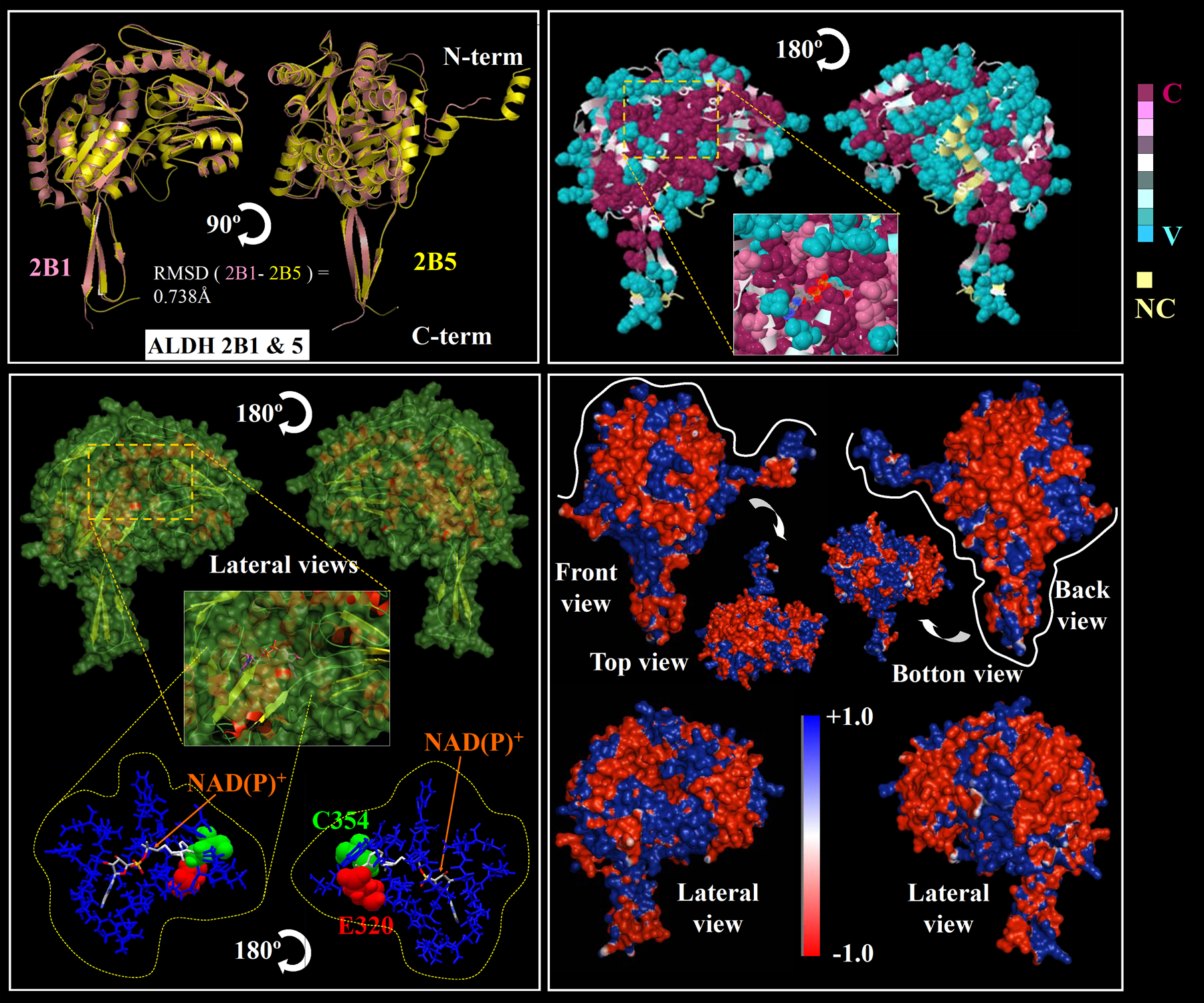

Supplement: Figure S2 — Detail structural conformation and conservation analysis of selected members of rice ALDH family 2, OsALDH2B1 and OsALDH2B5. (A) General structure (cartoon diagram) of the superimposition of OsALDH2B1 (light pink) and 2B5 (yellow) with RMSD calculated for each superimposition is shown. Represented structures were rotated at 90°. (B) Best predicted ALDH2B5 model (2D-structure) was subjected to consurf conservational analysis searching for close homologous sequences with known protein structures using PSI-BLAST. The protein was finally visualized using FirstGlance in Jmol, with the conservation scores colour-coded onto its surface. The conserved and variable residues are presented as a space-filled model, and coloured according to the conservation scores. A detailed view of the cavity holding up the NAD(P)+ cofactor (stick model and van der Walls spheres) is shown in high magnification. (C) Surface conformation of ALDH2B5 lateral views (rotated 180°), showing the secondary structure elements inside is depicted. The morphology of the cavity accommodating the cofactor is represented in high magnification. Detailed organization of the amino acid (aa) residues of the pocket is represented in blue. Stick model of the cofactor, and the catalytic aa residues (Cys 354 [green] and Glu 320 [red]), at opposite positions are shown. (D) Electrostatic surface potential showing all possible views of the ALDH2B5 structure. The surface colours are clamped at red (−1) or blue (+1). Top and bottom views are highlighted with a white line coming from the front view. (8.13 MB TIF) [file pone.0011516.s004.tif]

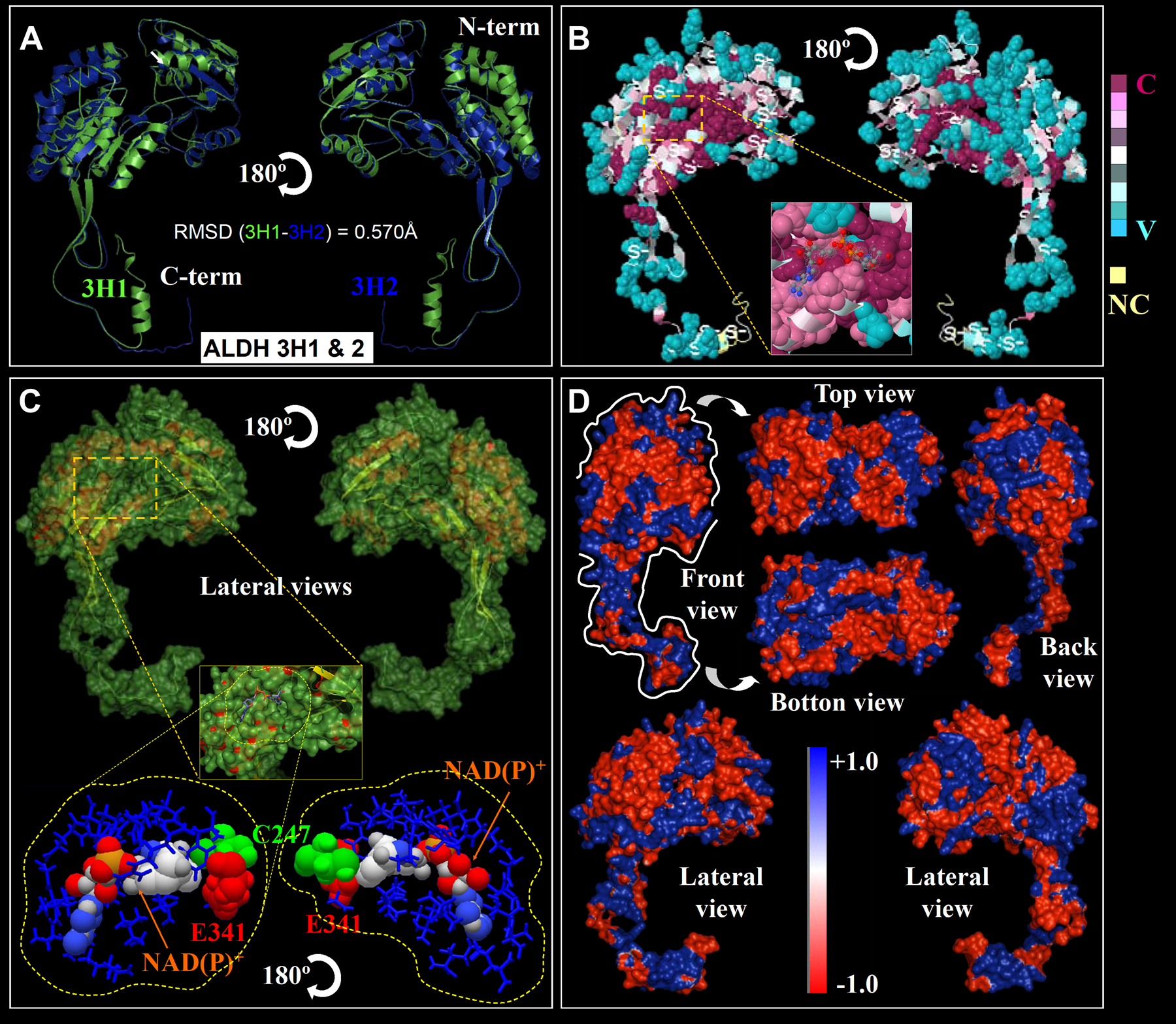

Supplement: Figure S3 — Detail structural conformation and conservation analysis of selected members of rice ALDH family 3, OsALDH3H1 and 3H2. (A) General structure (cartoon diagram) of the superimposition of OsALDH3H1 (green) and 3H1 (blue) with RMSD calculated for each superimposition is shown. Represented structures were rotated at 180°. (B) The best predicted ALDH3H2 model (2D-structure) was subjected to consurf conservational analysis searching for close homologous sequences of the protein of known structures using PSI-BLAST. The protein was visualized using FirstGlance in Jmol, with colour-coded conservation scores of its surface. The variable and conserved residues are presented as a space-filled model, and coloured according to the conservation scores. A detailed view of the cavity holding up the NAD(P)+ cofactor (stick model and van der Walls spheres) is shown. (C) Surface conformation of the ALDH3H2 lateral views (rotated 180°) is depicted showing the secondary structure elements inside. The morphology of the cavity accommodating the cofactor is represented in high magnification. Detail view organization of the predicted amino acids (aa) of the pocket is represented in blue colour. Space-filled representation of van der Waals surface of the cofactor, and the catalytic opposite positioned aa Cys 247 (green) and Glu 341 (red) is shown. (D) Electrostatic surface potential showing all the possible views of the ALDH3H2 structure. The surface colours are clamped at red (−1) or blue (+1). Top and bottom views are highlighted with a white line coming from the front view. (8.50 MB TIF) [file pone.0011516.s005.tif]

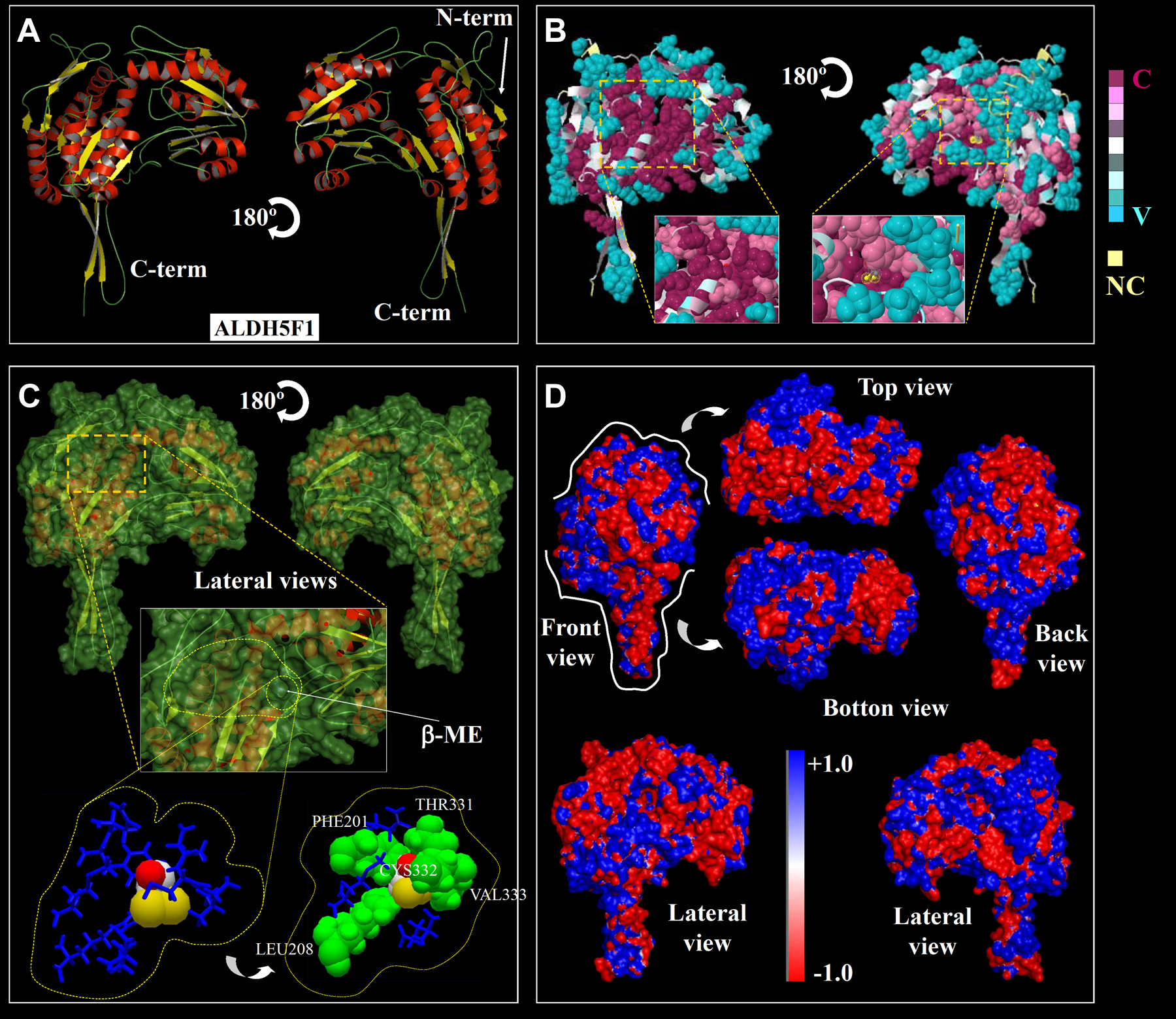

Supplement: Figure S4 — Detail structural conformation and conservation analysis of rice ALDH family 5 (ALDH5F1). (A, B, D) structural descriptions are similar to that of Figure S3 with exception of superimposition. (C) Detail view organization of the predicted amino acids (aa), which are close to the chemical ligand β-mercaptoethanol (β-ME) and the NAD(P)+ cofactor is represented in blue colour. Space-filled representation of van der Waals surface of β-ME, and the interacting aa Cys 332, VAL333, THR331, PHE201 and LEU208 are shown in green colour. (8.45 MB TIF) [file pone.0011516.s006.tif]

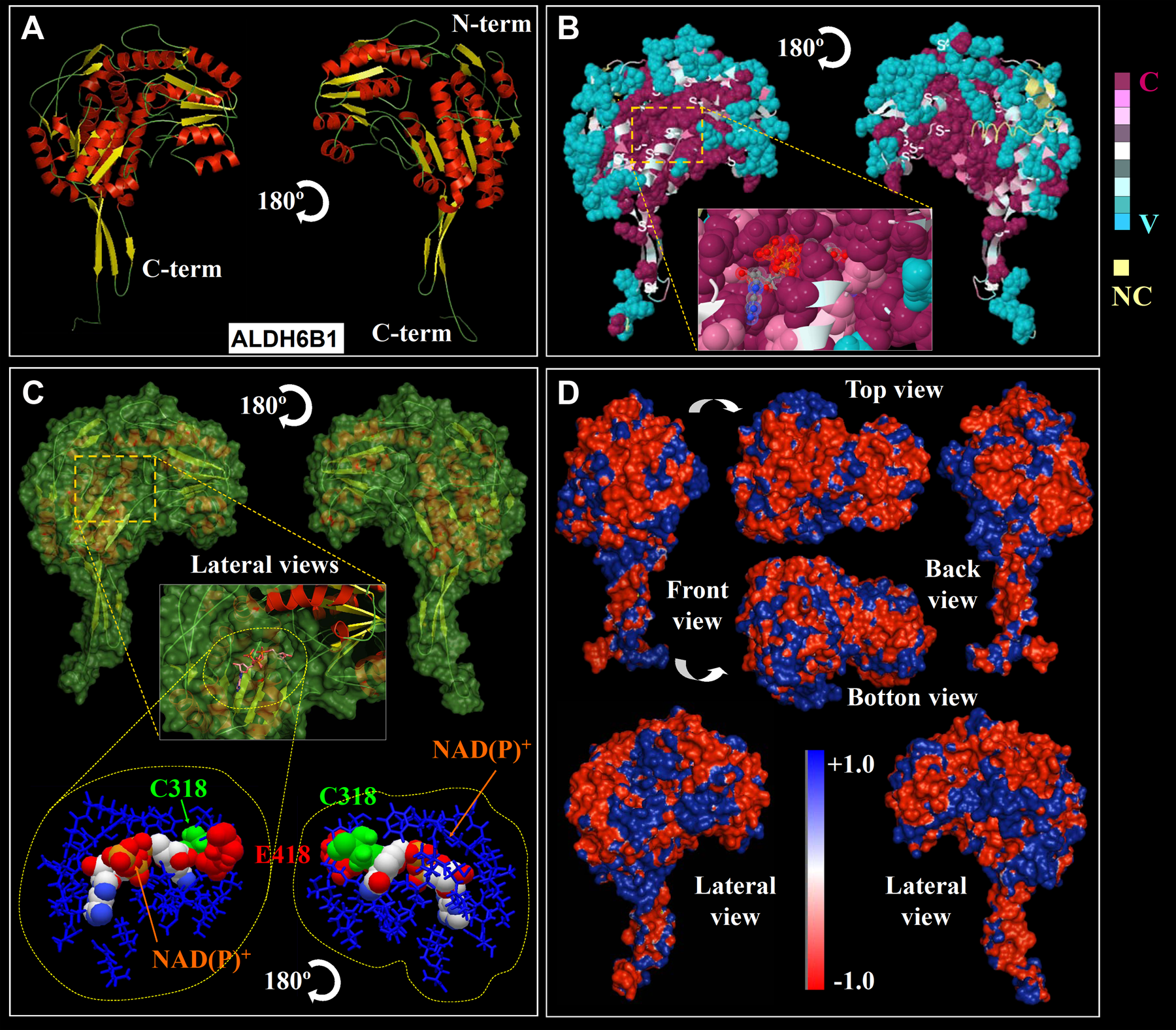

Supplement: Figure S5 — Detail structural conformation and conservation analysis of rice ALDH family 6 (ALDH6B1). (A, B, D) Detail description similar to that of Figure S3 with the exception of superimposition. The secondary structure elements are depicted in different colours: α-helix (red), β-sheet (yellow) and coils (green). (C) Space-filled representation of van der Waals surface of the cofactor, and the catalytic opposite positioned amino acids Cys 318 (green) and Glu 418 (red) are here shown. (8.55 MB TIF) [file pone.0011516.s007.tif]

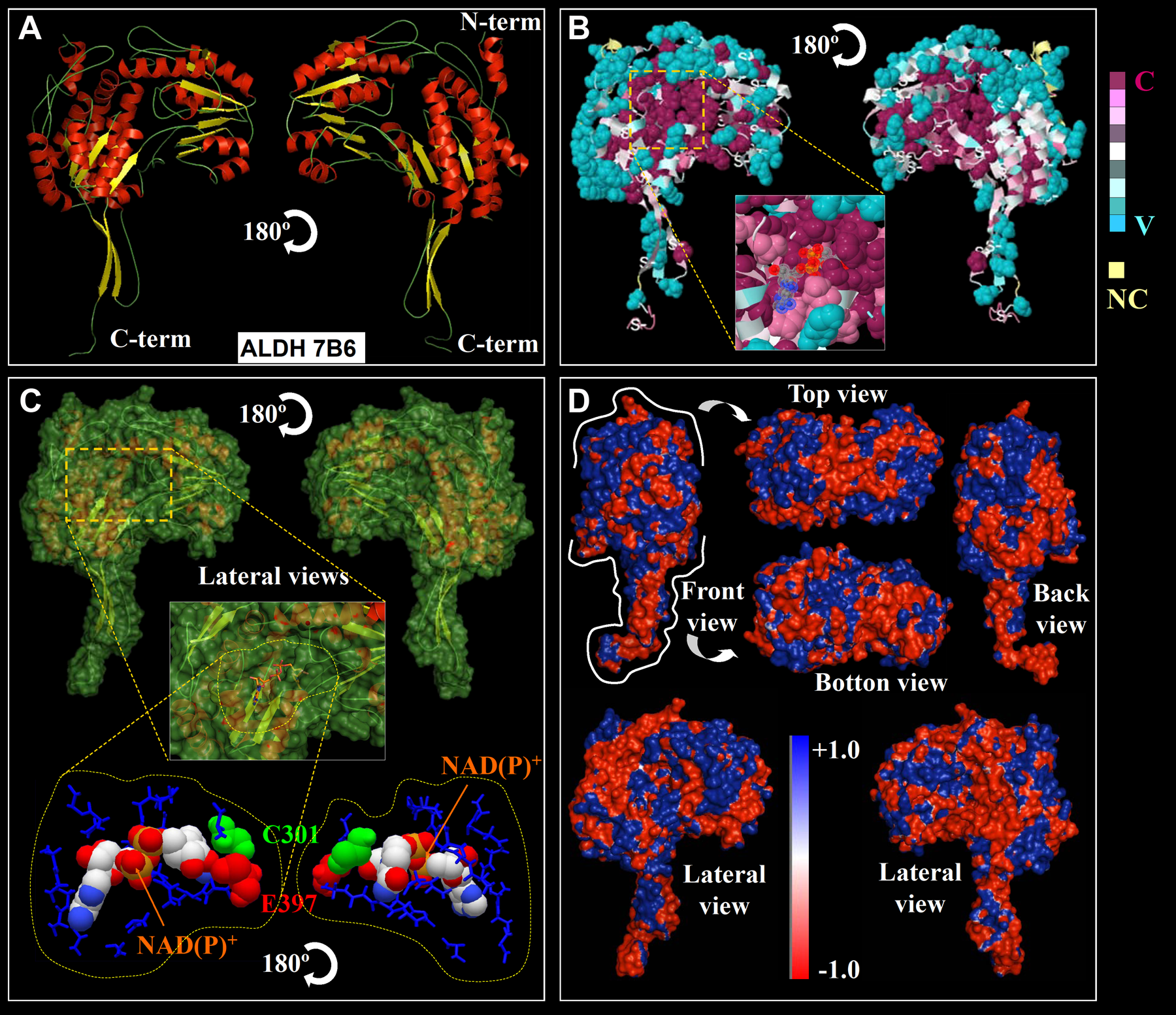

Supplement: Figure S6 — Detail structural conformation and conservation analysis of rice ALDH family 7 (ALDH7B6). (A, B, D) The structural description is similar to that of Figure S3 with the exception of superimposition. The secondary structure elements (A) are shown in different colours: α-helix (red), β-sheet (yellow) and coils (green). (C) The space-filled representation of van der Waals surface of the cofactor, and the catalytic opposite positioned amino acids Cys 301 (green) and Glu 397 (red) are shown. (8.42 MB TIF) [file pone.0011516.s008.tif]

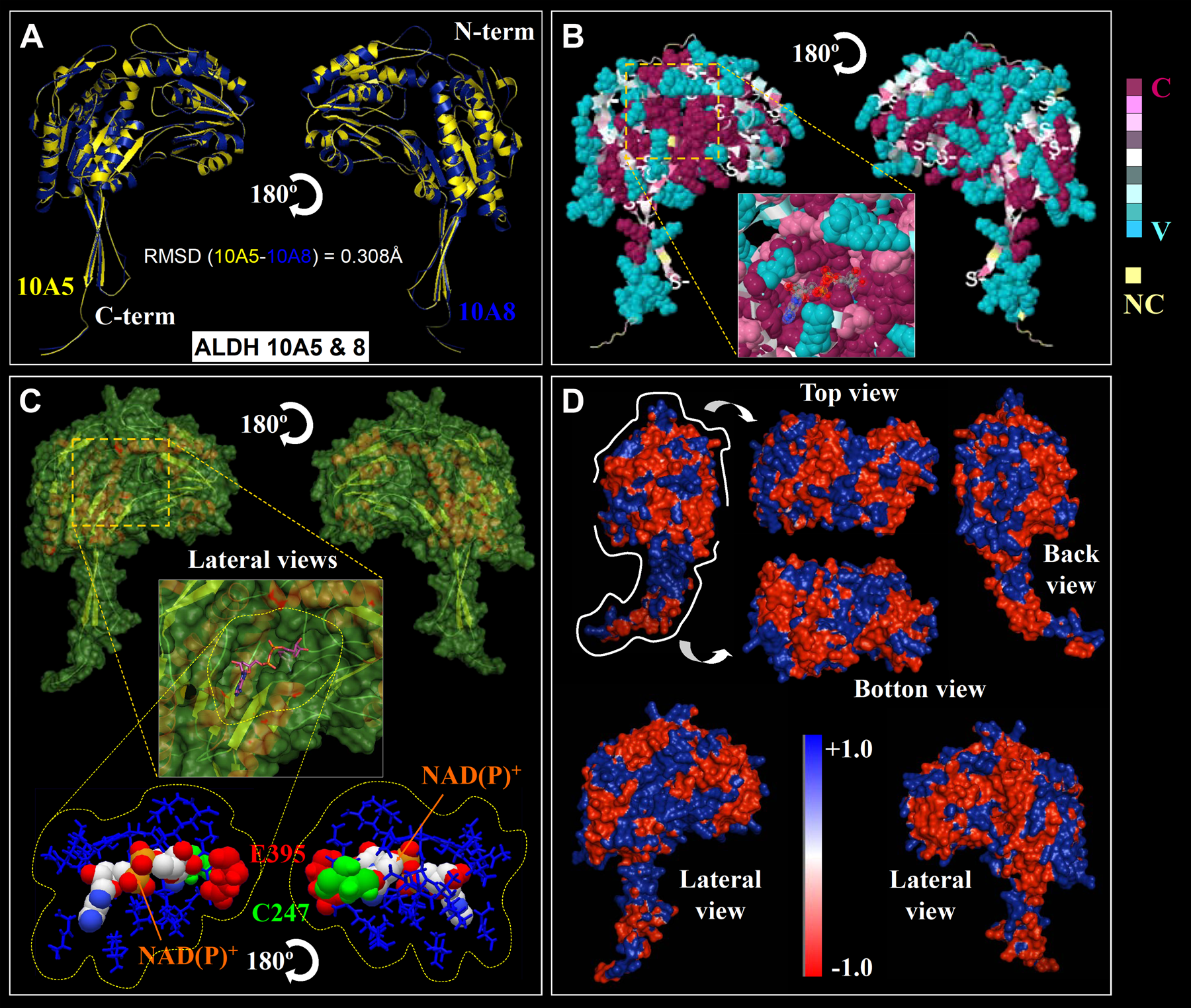

Supplement: Figure S7 — Detail structural conformation and conservation analysis of rice ALDH family 10 (ALDH10A5 and 10A8). (A, B, C) The structural description is similar to that of Figure S3. For the superimposition (A), OsALDH10A5 is represented in yellow and OsALDH10A8 is in blue. (C) The space-filled representation of van der Waals surface of the cofactor, and the catalytic opposite positioned amino acids Cys 247 (green) and Glu 395 (red) are here depicted. (8.26 MB TIF) [file pone.0011516.s009.tif]

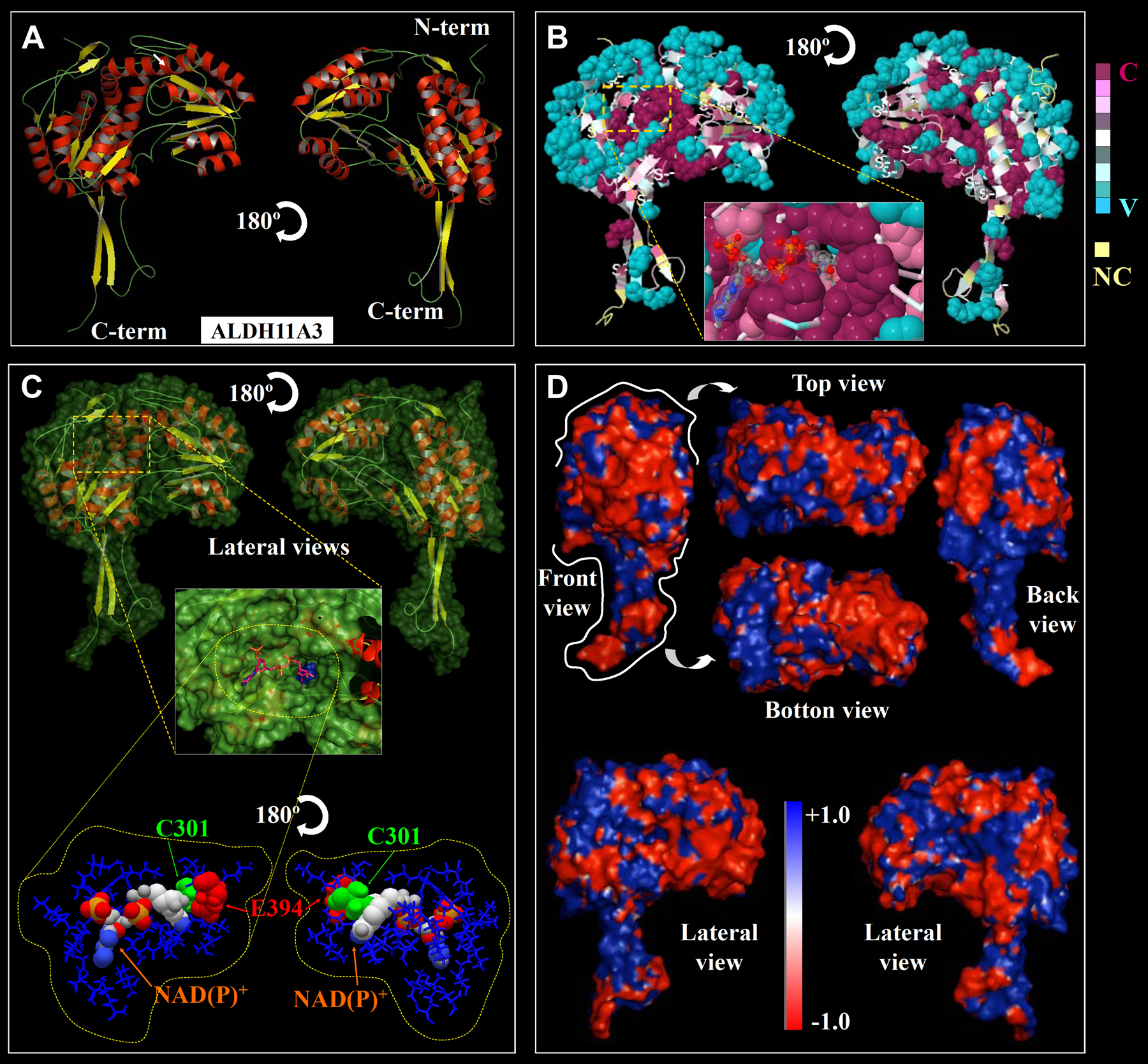

Supplement: Figure S8 — Detail structural conformation and conservation analysis of rice ALDH family 11 (ALDH11A3). (A, B, D) The structural description is similar to that of Figure S3 with the exception of superimposition. The secondary structure elements (A) are shown in different colours: α-helix (red), β-sheet (yellow) and coils (green). (C) The space-filled representation of van der Waals surface of the cofactor, and the catalytic opposite positioned amino acids Cys 301 (green) and Glu 394 (red) are shown. (9.04 MB TIF) [file pone.0011516.s010.tif]

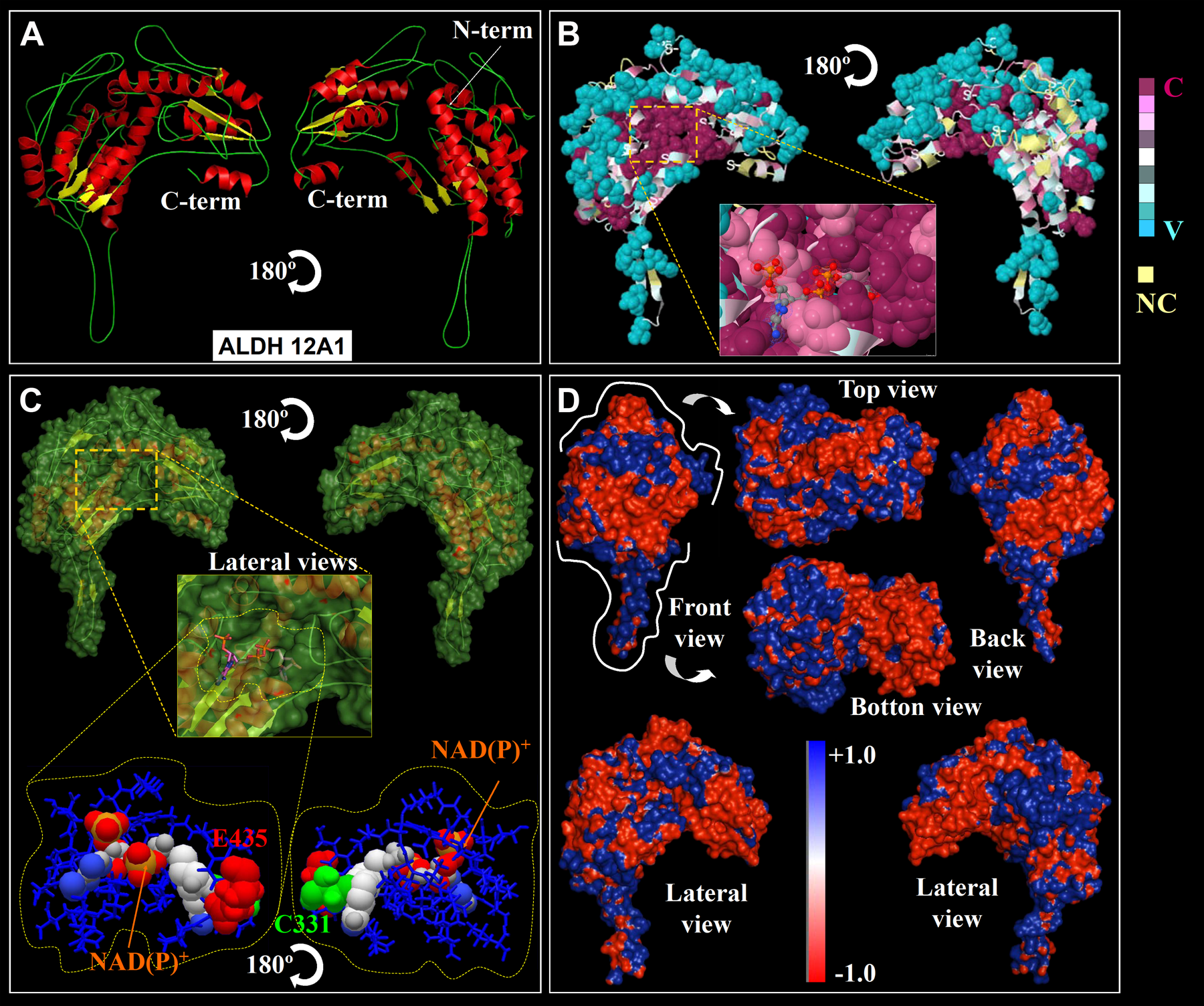

Supplement: Figure S9 — Detail structural conformation and conservation analysis of rice ALDH family 12 (ALDH12A1). (A, B, D) The structural description is similar to that of Figure S3 with the exception of superimposition. The secondary structure elements (A) are shown in different colours: α-helix (red), β-sheet (yellow) and coils (green). (C) The space-filled representation of van der Waals surface of the cofactor, and the catalytic opposite positioned amino acids Cys 331 (green) and Glu 435 (red) are shown. (8.15 MB TIF) [file pone.0011516.s011.tif]

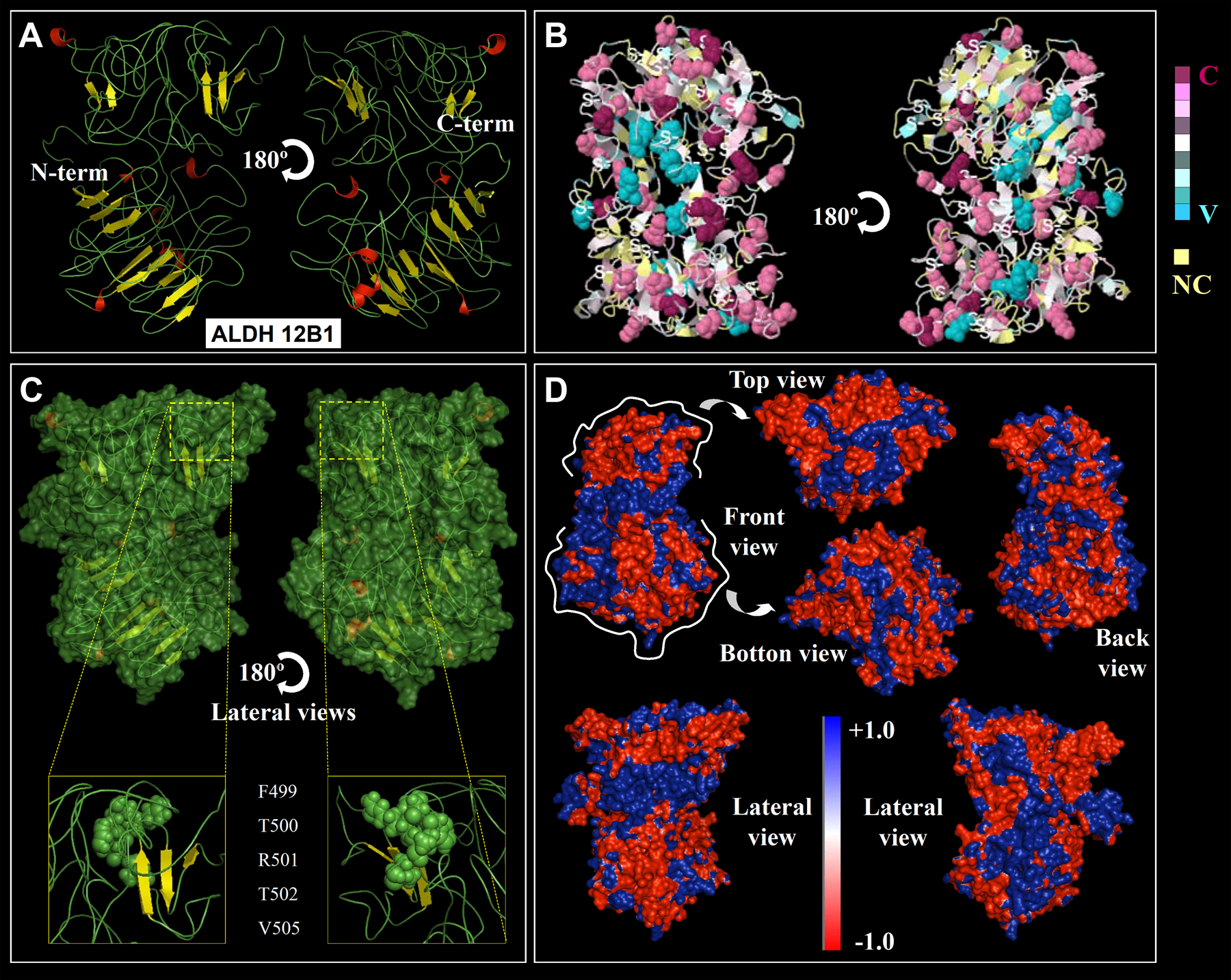

Supplement: Figure S10 — Detail structural conformation and conservation analysis of rice ALDH family 12 (ALDH12B1). (A, B, D) The structural description is similar to that of Figure S3 with the exception of superimposition. The secondary structure elements (A) are shown in different colours: α-helix (red), β-sheet (yellow) and coils (green). (C) The space-filled representation of van der Waals surface (in green colour) is represented, and the predicted amino acids comprising the pocket, which accommodate the NAD(P)+ cofactor F499, T500, R501, T502 and V505, are shown. (7.77 MB TIF) [file pone.0011516.s012.tif]

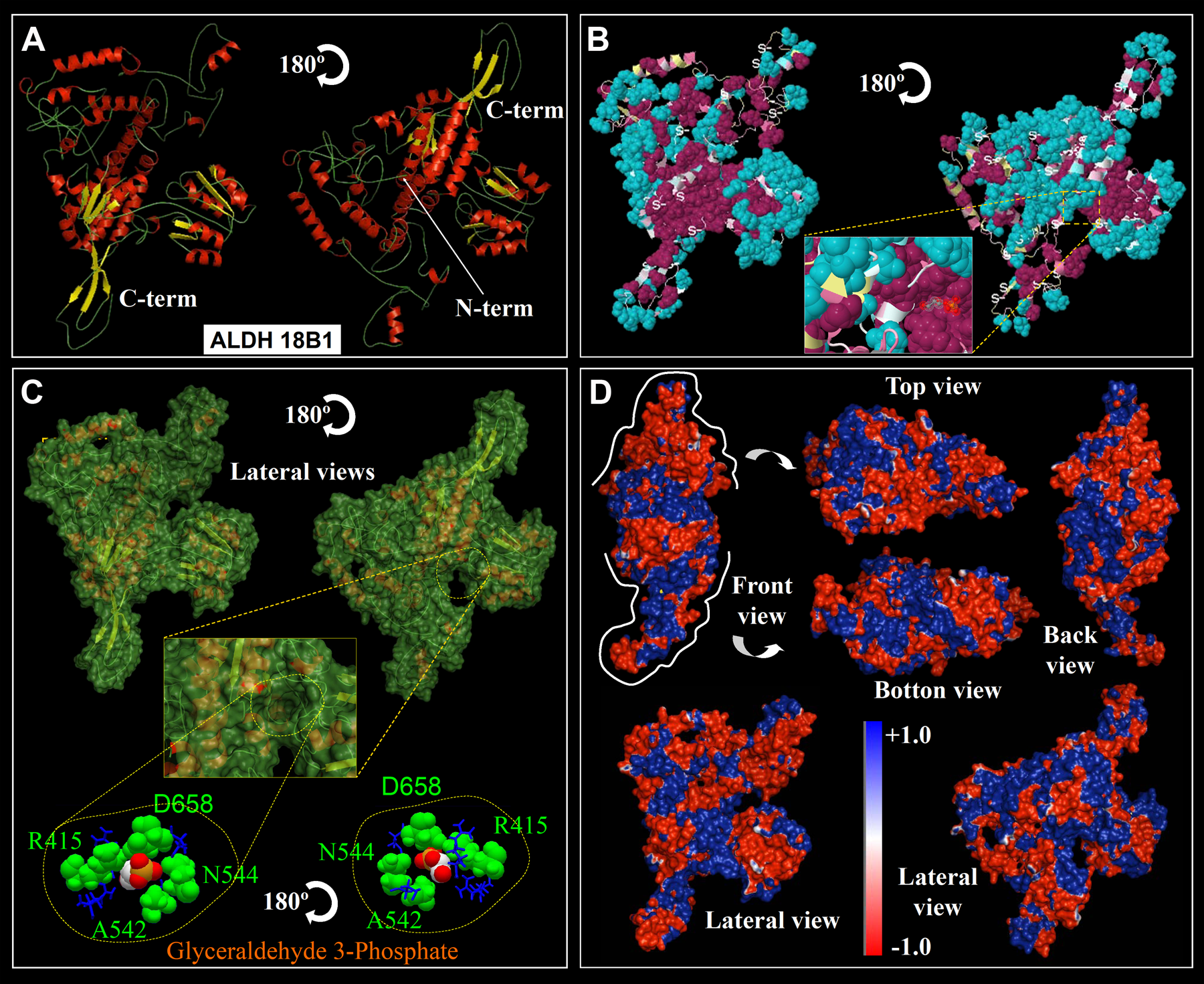

Supplement: Figure S11 — Detail structural conformation and conservation analysis of rice ALDH family 18 (ALDH18B1). (A, B, D) The structural description is similar to that of Figure S3 with the exception of superimposition. The secondary structure elements (A) are shown in different colours; α-helix (red), β-sheet (yellow) and coils (green). (B) A detailed view of the cavity holding up the molecule glyceraldehyde 3-Phosphate (stick model and van der Walls spheres) is shown. (C) Detail view organization of the predicted amino acids (aa) close to the chemical ligand glyceraldehyde 3-phosphate and the NAD(P)+ cofactor is depicted in blue colour. The space-filled representation of van der Waals surface of the molecule glyceraldehyde 3-Phosphate, and the interacting aa D658, N544, A542 and R415 are shown in green colour. (7.98 MB TIF) [file pone.0011516.s013.tif]

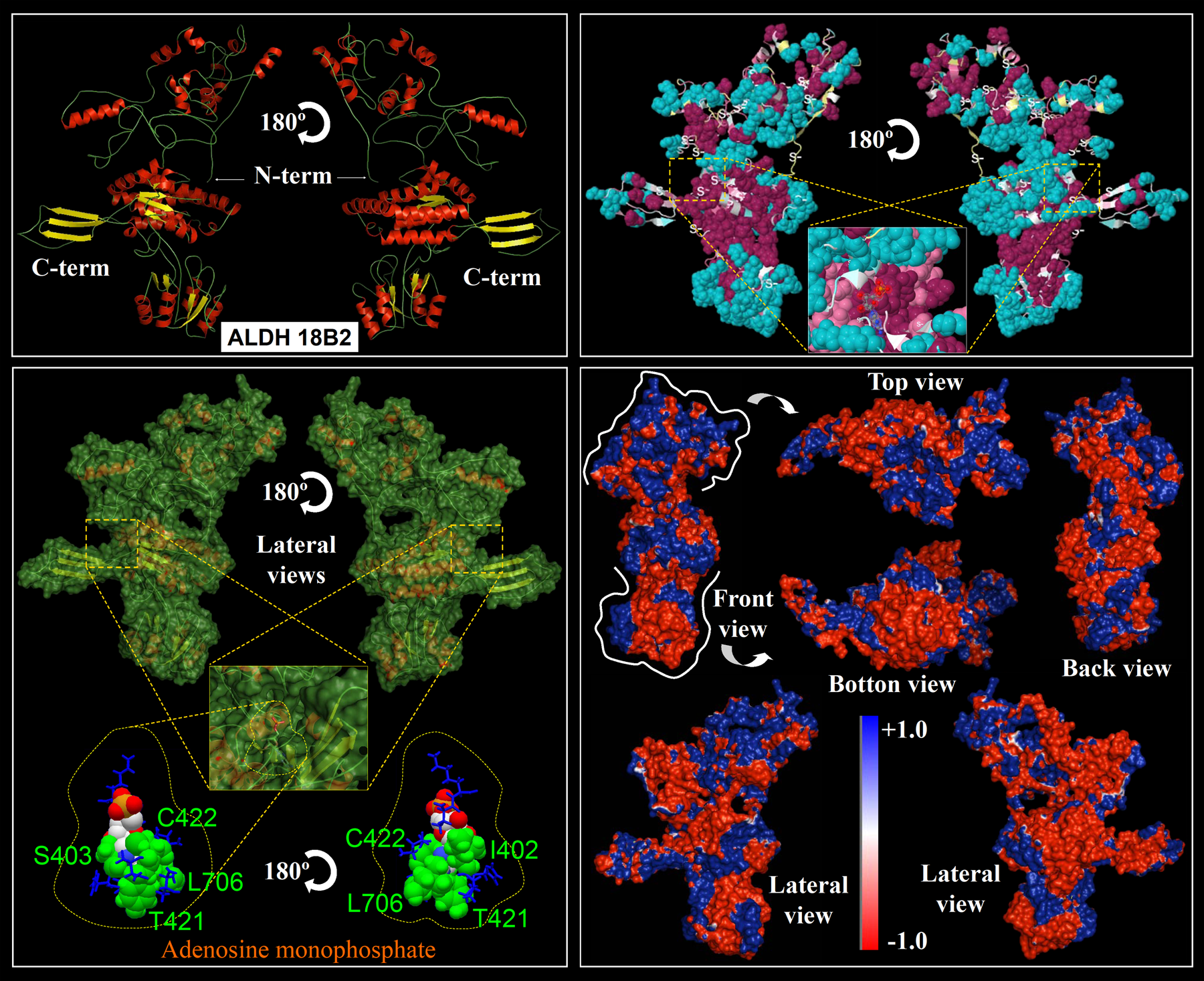

Supplement: Figure S12 — Detail structural conformation and conservation analysis of rice ALDH family 18 (ALDH18B2). (A, B, D) The structural description is similar to that of Figure S3 with the exception of superimposition. The secondary structure elements (A) are shown in different colours: α-helix (red), β-sheet (yellow) and coils (green). (C) Detail view organization of the predicted amino acids (aa) close to close to the chemical ligand adenosine monophosphate and the NAD(P)+ cofactor is shown in blue colour. Space-filled representation of van der Waals surface of the molecule adenosine monophosphate, and the interacting aa S403, T421, C422 and L706 are shown in green colour. (7.95 MB TIF) [file pone.0011516.s014.tif]

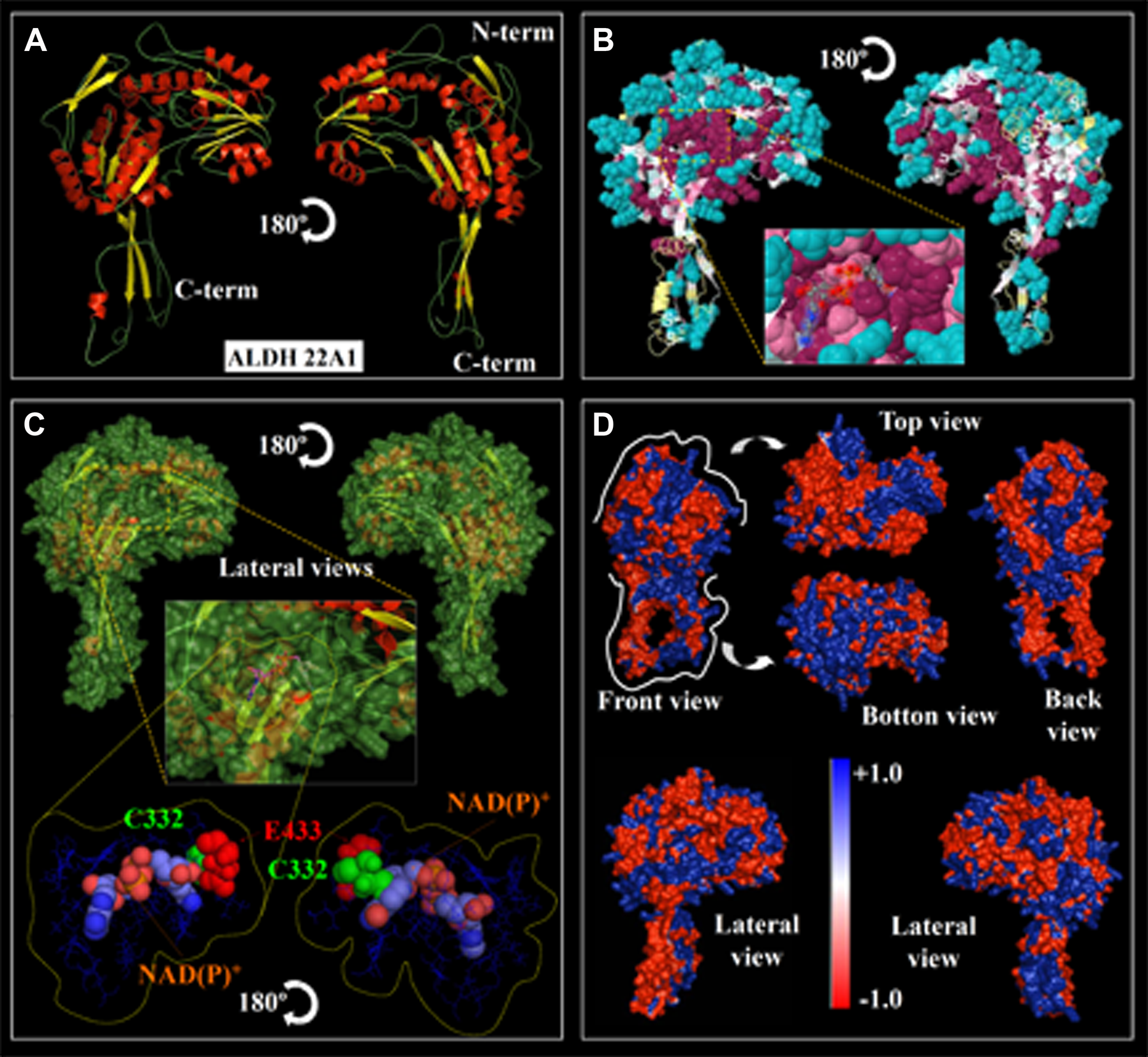

Supplement: Figure S13 — Detail structural conformation and conservation analysis of rice ALDH family 22 (ALDH22A1). (A, B, D) The structural description is similar to that of Figure S3 with the exception of superimposition. The secondary structure elements (A) are shown in different colours: α-helix (red), α-sheet (yellow) and coils (green). (C) The space-filled representation of van der Waals surface of the cofactor, and the catalytic opposite positioned amino acids Cys 332 (green) and Glu 433 (red) are shown. (8.99 MB TIF) [file pone.0011516.s015.tif]
